# Toll-like receptor adaptor protein TIRAP has specialized roles in signaling, metabolic control and leukocyte migration upon wounding in zebrafish larvae

**DOI:** 10.7150/ijbs.101055

**Published:** 2025-01-01

**Authors:** Li Liu, Wanbin Hu, Fatima Didar Kerman, Herman P. Spaink

**Affiliations:** 1 Institute of Biology Leiden, Animal Science and Health, Leiden University, Einsteinweg 55, 2333 CC Leiden, The Netherlands.; 2 Present address: Center for Thrombosis and Hemostasis (CTH), Johannes Gutenberg-University Medical Center, Langenbeckstraße 1, Bldg. 70855131 Mainz, Germany.

**Keywords:** tirap, Mal, transcriptome, metabolome, neutrophil migration, tissue wounding

## Abstract

The TIRAP protein is an adaptor protein in TLR signaling which links TLR2 and TLR4 to the adaptor protein Myd88. The transcriptomic profiles of zebrafish larvae from a* tirap*,* myd88* and* tlr2* mutant and the corresponding wild type controls under unchallenged developmental conditions revealed a specific involvement of *tirap* in calcium homeostasis and myosin regulation. Metabolomic profiling showed that the *tirap* mutation results in lower glucose levels, whereas a *tlr2* mutation leads to higher glucose levels. A tail-wounding zebrafish larval model was used to identify the role of *tirap* in leukocyte migration to tissue wounding. We found that more neutrophils were recruited to the wounded region in the *tirap* mutant larvae compared to the wild type controls, whereas there was no difference in macrophage recruitment. In contrast, published data show that *tlr2* and *myd88* mutants recruit fewer neutrophils and macrophages to the wounds. Based on cell tracking analysis, we demonstrate that the neutrophil migration speed is increased in the *tirap* mutant in contrast to neutrophil behavior in *myd88* and* tlr2* mutants. In conclusion, we show that *tirap* plays specialized roles distinct from *tlr2* and *myd88* in signaling, metabolic control, and in regulating neutrophil migration speed upon wounding.

## 1. Introduction

Membrane-localized pattern recognition receptors (PRRs) are required by leukocytes during perception and recognition processes facing invasive infection and tissue damage^1^, ^2^. Toll-like receptors (TLRs) are among the most extensively studied PRRs and are the crucial recognition factors for pathogen-associated molecular patterns (PAMPs) derived from invading pathogens and damage-associated molecular patterns (DAMPs) released by stressed, damaged or dead cells^3^. Upon interaction with PAMPs and DAMPs, TLRs can bind to some adapters to initiate downstream signaling cascades. The Toll-interleukin-1 receptor (TIR) domain-containing adaptor protein (TIRAP), also known as MyD88-associated ligand (MAL), is an adaptor protein of TLR2 or TLR4^4^, ^5^. As a key intracellular signaling transducer, TIRAP contains a TIR domain that can bind to the TIR domain of TLR2 and then recruits MyD88^6^. TIRAP thereby activates the downstream cascade of MyD88-dependent TLR signaling, ultimately leading to the production of proinflammatory cytokines and in this way is involved in the regulation of the innate immune responses of leukocytes^7^, ^8^. It is therefore crucial to understand the underlying signaling mechanisms mediated by the TLRs pathway and the modulatory roles of TIRAP within it.

TLR signaling has been recently shown to be involved in the control of migration of leukocytes towards tissue damage and wounding. Leukocytes can be rapidly recruited to the site of injury by inflammatory mediators and chemokines, and play a crucial role in immune clearance and damage repair upon tissue wounding^9^. As first responders to wounded tissues, the recruitment and migration of leukocytes to injured tissues is particularly important in the acute inflammatory response^10^. In our previous study on TLR signaling influencing leukocyte migration in a zebrafish larval model, it was found that both *tlr2* and *myd88* mutants recruited less neutrophils and macrophages to the wounding area than their wild type controls^11^. However, the role that TIRAP plays in this process has not been studied.

Since the first discovery of TIRAP in 2001, the function of TIRAP in diverse immune responses as an adaptor molecule has gradually been revealed^12^, ^13^. Studies in lung diseases have demonstrated the important role of TIRAP in immune modulation by affecting cytokines and chemokines. For example, when TIRAP is deficient, the lipopolysaccharide (LPS) induced expression of cytokines and chemokines such as tumor necrose factor-α (TNF-α), interleukine-6 (IL-6) and IL-8 in alveolar macrophages is attenuated^14^. Also, macrophages from TIRAP mutant mice were defective in the early burst of proinflammatory cytokine production and in the ability to limit intracellular growth of pathogen *Bordetella pertussis*^15^. Studies in inflammatory signaling have further revealed that the regulated activation of TIRAP is an important part of preventing excessive inflammatory damage to the host^8^. Tyrosine kinases such as Bruton's tyrosine kinase (BTK) can tyrosine phosphorylate the TIR domain of TIRAP, therefore transiently activating TIRAP, and suppressor of cytokine signaling 1 (SOCS1) can mediate the ubiquitin-dependent degradation of phosphorylated TIRAP^16^. The transient activation of TIRAP and subsequent SOCS-1-mediated degradation leads to a rapid and balanced inflammatory response^16^. The normal function of TIRAP is thereby essential to avoid contributing to chronic inflammation and related diseases^16^. There is also evidence that TIRAP can be recruited by the receptor for advanced glycation end products (RAGE) and such engagement allows for the activation of downstream effector molecules that mediate various cellular processes including inflammatory responses and apoptotic cell migration and death^17^, ^18^. Mutations in the TIR domain of TIRAP lead to the inhibition of downstream inflammatory signaling, as well as affecting the role of RAGE in inducing proinflammatory immune responses during disease^17^, ^18^. TIRAP enables interaction with various intracellular signaling molecules and influences leukocyte immune function, indicating its central role in diverse immune responses. However, current research implies that the role of TIRAP in the resolution of inflammation is a double-edged sword^19^ and that the impact on the immune response still needs further study, as does the function of TIRAP in the migratory behavior of immune cells.

Current advances in live imaging and the application of new model systems have contributed to revealing the motility behavior of leukocyte cells in wounded and infected tissues. As the small size and transparency of zebrafish larvae are useful features for screening and imaging transgenic reporter strains, zebrafish larvae are considered an ideal model for visualizing and tracking fluorescent leukocytes in the resolution of inflammation such as bacterial infection and tail wounding^20^, ^21^. Some signals have been defined to influence leukocyte motility behavior in the resolution of inflammation through the application of zebrafish models. For example, the chemokine receptors C-X-C Motif Chemokine Receptor 1(Cxcr1) and Cxcr2 can regulate the recruitment of neutrophils to the sites of tissue damage in zebrafish larvae^22^. In our previous study, also using a tail-wounding zebrafish larval model, the importance of Tlr2 and its adaptor protein Myd88 in the regulation of leukocyte migration in response to tail wounding has been revealed. This confirmed the involvement of TLR signaling in controlling the migration behavior of neutrophils and macrophages during wounding^11^.

In order to explore the specific role of Tirap, we systematically investigated the transcriptional and metabolic profiles using RNAseq and ^1^H nuclear magnetic resonance (NMR) techniques in *tirap* mutant and also in *myd88* mutant fish lines. We compared our results with data from the *tlr2* mutant from our previous study^23^. We found that significantly differentially expressed genes in the *tirap* mutant are different from *myd88* and *tlr2* mutants. In addition, fewer differentially altered metabolites were found in the *tirap* mutant than in the *tlr2* mutant. This implies that* tirap* is different from *tlr2* and *myd88* in terms of transcriptional level and metabolic regulation. To investigate the regulatory role of *tirap* in the innate immune process in cellular responses to inflammation, we used live fluorescent imaging to study the response after mycobacterial infection and leukocyte migration after tail wounding in larvae. In the infection experiments, *tirap* mutant larvae had a higher bacterial burden after infection with *Mycobacterium marinum* strain Mma20 than the wild type control. In the tail wounding experiments, *tirap* mutant larvae recruited significantly more neutrophils to the wounds than the wild type control, whereas there was no significant difference in the recruitment for macrophages. Further studies of neutrophil motility showed that *tirap* controls neutrophil migration speed, but not directional persistence upon tail wounding. Finally, we summarize the results of all our zebrafish larval test systems with mutants of *tirap*, *myd88* and *tlr2*.

## 2. Materials and Methods

### 2.1. Zebrafish maintenance and mutant line construction

The maintenance of all adult and larval zebrafish and all animal experiments described in this study were conducted at Leiden University in accordance with the standard protocols (zfin.org) and adhered to the international guidelines specified by the EU Animal Protection Directive 2010/63/EU. The culture of adult fish was approved by the university's local animal welfare committee (DEC) (License number: protocol 14,198) and no adult zebrafish were sacrificed in this study. Eggs and larvae were grown in laboratory-manufactured egg water (containing 60 mg/l instant ocean sea salts) at 28.5℃. For infection, wounding and live imaging assays, the experiments were all performed on larvae up to 5 days post fertilization (dpf) and therefore prior to the free-feeding stage, which is not covered by the animal experimentation law according to the EU Animal Protection Directive 2010/63/EU. 27 and 28 hours post fertilization (hpf) larvae during injection experiments and 3 dpf larvae during wounding experiments were anesthetized with egg water containing 0.02% buffered 3-aminobenzoic acid ethyl ester (Tricaine, Sigma-Aldrich, Netherlands).

The *tirap^sa182^* mutant (further referred as *tirap^-/-^* or *tirap* mutant) line (ENU-mutagenized) was obtained from the Sanger Institute Zebrafish Mutation Resource (Hinxton, Cambridge, UK) and shipped by the zebrafish resource Center of the Karlsruche Institute of Technology. All mutant alleles were identified by sequencing and the homozygote carriers of the mutations were outcrossed more than three times to wild type (AB/TL strains). Homozygote mutants and their wild type siblings (further referred as *tirap^+/+^*) were used to generate models in this study. To investigate the effect of the *tirap* mutation on the cell migration behavior, fluorescent transgenic lines *tirap^+/+^ Tg* (*mpeg1*:* mCherry-F*), *tirap^+/+^ Tg* (*mpx*:* EGFP*), *tirap^-/-^ Tg* (*mpeg1*:* mCherry-F*) and *tirap^-/-^ Tg* (*mpx*:* EGFP*) were used. The generation of transgenic lines and *myd88* mutant zebrafish line refer to previous studies^11^, ^21^.

### 2.2. LPS injection and qRT-PCR detecting

In order to determine whether the *tirap* mutation results in a loss of function of Tirap and blocked the downstream pathway, we detected the downstream gene expression in zebrafish larvae after injection with lipopolysaccharide (LPS)^24^. 1 nl purified LPS (100 μg/ml) solutions were injected into the blood stream through the blood island at 27 hpf for each larva using a Femtojet microinjector (Eppendorf, the Netherlands) equipped with a capillary glass needle. 1 nl sterile water was injected as the control experiment. At 1 hour post injection (hpi), the injected larvae were collected for the quantitative real-time PCR (qRT-PCR) analysis. qRT-PCR were performed on a CFX96TM Touch Real-Time PCR Detection (Bio-Rad Laboratories, Inc, USA). The expression of target gene was normalized to the expression of *ppial* as a reference gene. The primer sequences used in this study are shown in Supplementary Table 1. The qRT-PCR reaction procedure was performed according to the following protocol: 95℃ 3 min, 40 cycles real time of 95℃ 15 sec, 68℃ 30 sec and 72℃ 30 sec, and final melting curve of 95℃ 1 min and 55℃ 10 sec. The quantitative qRT-PCR assay was biologically repeated for three times and the relative expression level was determined by the comparative 2^-ΔΔCt^ method^25^.

### 2.3. RNAseq processing and analysis

For the purpose of RNA sequencing, the extraction of total RNA from 5 dpf *tirap* mutant zebrafish larvae and wild type controls was performed using TRIzol Reagent (Life Technologies), following the manufacturer's instructions. DNase treatment was then conducted by using the kit (Thermo Scientific) to remove the DNA contamination. RNA sequencing of unchallenged *tirap* mutant and wild type zebrafish larvae was conducted by GenomeScan B.V. (Leiden, The Netherlands) as previously described^26^. Sequencing data of three biological replicates for the *tirap* mutants and wild type controls were aligned and mapped to the zebrafish genome GRCz11 using Salmon v1.2.1^27^. Differential gene expression was analyzed by using DESeq2 v1.24.0^28^. Statistical significance was determined by a padj value of ≤ 0.05^29^. KEGG pathway enrichment analysis was performed in DAVID Bioinformatics Resources 6.8 (https://david.Ncifcrf.Gov/). For unchallenged *myd88* mutant and wild type zebrafish larvae, consistent sequencing process and analysis were performed.

### 2.4. NMR sample preparation and measurement

The extraction of metabolites from zebrafish larvae in wild type and mutant groups was based on our previous research method^30^, ^31^. Each group contained 120 zebrafish larvae at 5 dpf in a mixture of methanol : water (1:1, v/v) with 1 ml of chloroform. The mixture was sonicated for 15 min and then centrifuged at 5,000 rpm for 5 min. After centrifugation, two layers were formed. The methanol and water from the upper layer was collected and then dried with nitrogen. The dried methanol : water layer containing metabolites was dissolved in 1 ml of 100 mM deuterated phosphate (KD_2_PO_4_, pH: 7.0) containing 0.02% trimethylsilyl propionate (TSP) as internal standard and then filtered using a Millipore filter (Millex-HV0.45-lmFilterUnit). Metabolites in zebrafish larvae were measured using a Bruker DMX 600 MHz NMR spectrometer at 4°C. The spectrometer was equipped with a 5 mm inverted triple high resolution probe with an actively shielded gradient coil. In order to obtain a satisfactory signal-to-noise ratio, the ^1^H NMR spectra were accumulated with 65,000 data points, a relaxation delay of 2 s, a scan width of 12.4 kHz and 256 scans.

One-dimensional (1-D) ^1^H NMR spectra obtained from both the wild type and mutant groups were corrected for baseline and phase shifts with MestReNova software version 11.0 (Mestrelab Research S.L., Santiago de Compostela, Spain). The spectra were then subdivided into buckets of 0.04 ppm in the range of 0 to 10.00 ppm. For removal of the water peak, the range from 4.30 to 6.00 ppm was excluded from the analysis. The spectra from chemical shifts 0.80 to 4.30 ppm were assigned to specific metabolites. Metabolite quantification was performed using Chenomx NMR Suite 8.3, which allows both qualitative and quantitative analysis of the NMR spectra by fitting spectral features from the HMDB database to the spectra. The data matrix obtained was exported to Microsoft Office Excel (Microsoft Corporation). These data were then simultaneously imported into MetaboAnalyst 4.0 for the Partial least squares-discriminant analysis (PLS-DA) and heat map analysis. A correlation coefficient of *P* < 0.05 was considered to be statistically significant.

### 2.5. Infection modelling

In the present study, a bacterial strain *Mycobacterium marinum m20* (Mma20) expressing mCherry fluorescent protein was used for infection experiments. The preparation of bacteria was performed as previously described^32^. The infection inoculum was prepared in 2% polyvinylpyrrolidone 40 solution (Calbiochem, the Netherlands), and approximately 150 colony-forming units (CFU) of bacteria were injected into the blood stream through the blood island at 28 hpf for each larva^26^. At 4 days post infection (dpi), pools of larvae were collected and imaged using the MZ16FA Fluorescence Stereo Microscope (Leica Microsystems, Wetzlar Germany). The statistical analysis of bacterial burden of the whole body of the larvae was conducted respectively in a dedicated pixel counting software QuantiFish 2.1 program^33^.

### 2.6. Tail wounding modelling and qRT-PCR detecting

The caudal fin wounding model in this study was applied based on previous studies^11^, ^21^. 3 dpf *tirap* mutant zebrafish larvae and their wild type controls were anesthetized in egg water with 0.02% tricaine (Sigma Aldrich). The inactive larvae were then placed in a 2% agarose covered petri dish and the caudal fins of larvae were wounded by using a 1 mm sterile sapphire blade scalpel (World Precision Instruments) under a MZ16FA Fluorescence Stereo Microscope (Leica Microsystems, Wetzlar Germany) equipped with a DFC420C color camera (Leica Microsystems). After the wounding, the wounded larvae were placed back into untreated egg water and incubated at 28.5 ℃ for the subsequent imaging experiments. We performed qRT-PCR on a CFX96TM Touch Real-Time PCR Detection (Bio-Rad Laboratories, Inc, USA) to quantify the immune related gene expression profiles from *tirap* wild type and mutant larvae after tail wounding. Target gene expression was normalized to the expression of *ppial* as a reference gene. The primer sequences used in this study are shown in Supplementary Table 1. The qRT-PCR reaction procedure and condition were the same as described in section 2.2.

### 2.7. Imaging and quantification

At 1, 3, and 6 hours post wounding (hpw), larvae were collected and fixed with 4% paraformaldehyde (PFA). The wounded tail area of each fixed specimen was imaged by using the Leica MZ16FA fluorescence stereo microscope. The number of neutrophils and macrophages recruited to the wounded area after tail wounding was manually quantified. Cells localized within an area of 200 µm from the wounding edge toward the body trunk were counted as recruited cells^11^.

### 2.8. Live imaging and quantification

Time lapse imaging of 3 dpf zebrafish larvae were visualized under a Leica TCS SP8 confocal laser scanning microscope (CLSM, Leica Microsystems, Wetzlar Germany) with 1 min time interval for 2 h using a 20× objective (N.A.0.75). For unchallenged condition, the caudal hematopoietic tissue (CHT) of zebrafish larvae was imaged using the CLSM. For tail wounding condition, the wounded tail area of zebrafish larvae was imaged from 1 hpw to 3 hpw under the CLSM. The quantification for leukocyte cell behavior was conducted in IMARIS x64 7.4 Software program (Bitplane), which loaded with an automated 3D cell tracking algorithm was employed to establish neutrophil trajectories in live imaging of zebrafish larvae. Using the IMARIS software, data of the number, mean speed, and meandering index of recruited neutrophils in the wounded tail region were obtained.

### 2.9. Statistical analysis

The statistical analysis was performed using the Graphpad Prism software (Version 8.1.1; GraphPad Software, San Diego, CA, USA). All experimental data in this study are shown as mean ± SD. D'Agostino-Pearson and Shapiro-Wilk normality test was performed to determine the Gaussian distribution of the data. The statistical significant differences between wild type and *tirap* mutant larvae were determined based on the Gaussian distribution of data using either Mann-Whitney and Kolmogorov-Smirnov (non-parametric tests) or unpaired t test with Welch's correction (parametric tests). The significance was established as * *P* < 0.05, ** *P* < 0.01 and *** *P* < 0.001.

## 3. Results

### 3.1. Characterization of tirap mutant zebrafish line

**Figure 1:**
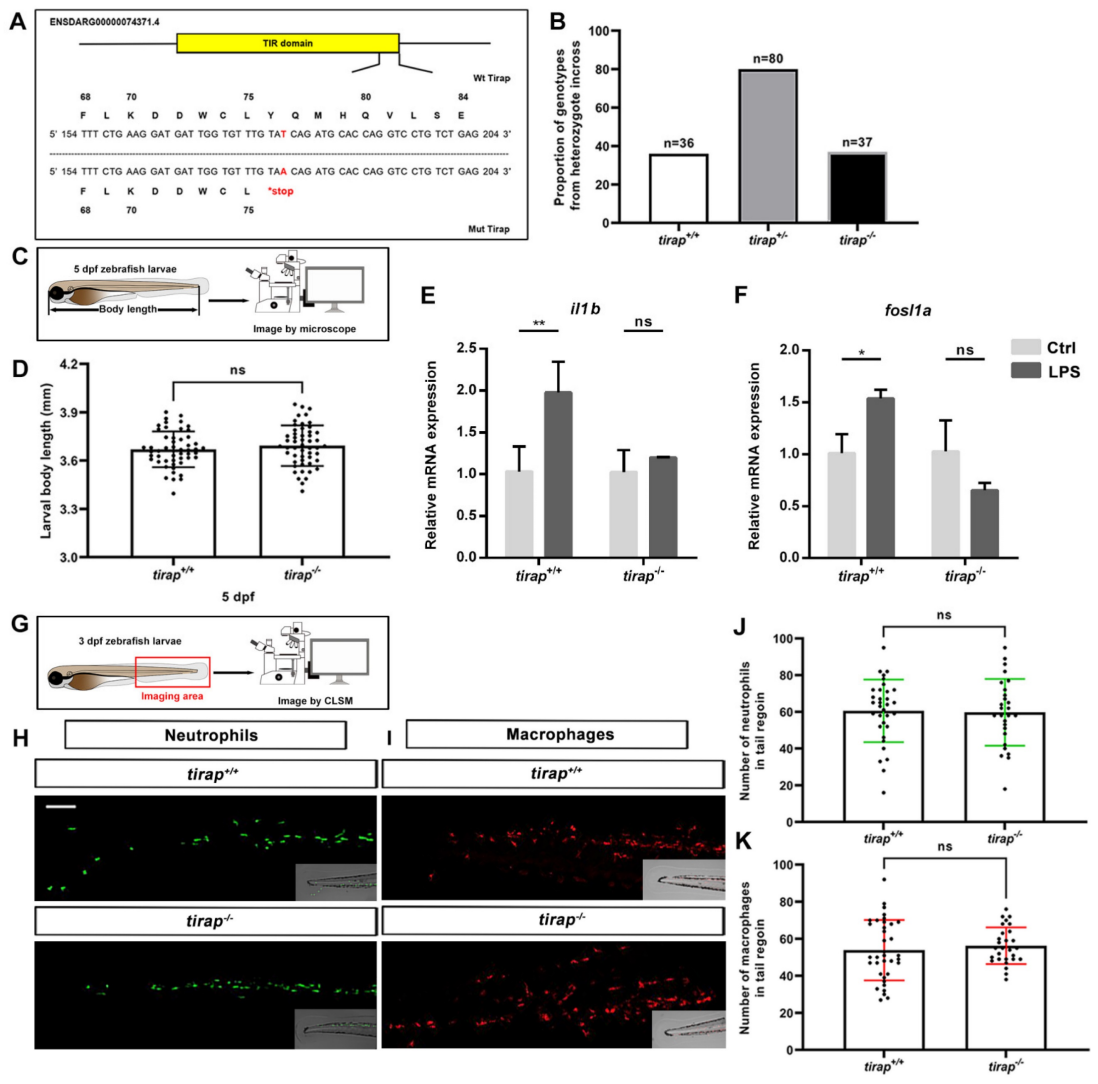
Characterization of *tirap* mutant zebrafish larvae. (A) Mutant DNA and protein sequence of Tirap. A point mutation (T to A) in the TIR domain of zebrafish *tirap* results in a stop codon. (B) Proportion of genotypes. The proportion of genotypes after the incross of *tirap^+/-^* heterozygous. (C) Experimental scheme of body length measurement. (D) Development. The body measurement of *tirap^+/+^* and *tirap^-/-^* zebrafish at 5 days post fertilization (5 dpf). The measurements are based on 3 independent experiments with *tirap^+/+^* (n=52) and *tirap^-/-^* (n=52). (E, F) qPCR of *tirap^+/+^* and *tirap^-/-^* zebrafish embryos. *Tirap^+/+^* and *tirap^-/-^* embryos were injected with purified LPS (100 µg/ml) or sterile water as a control at 27 hour post fertilization (hpf). The expression level of *il1b* and *fosl1a* were analyzed at 1 hour post injection (hpi) by qRT-PCR. Data (mean ± SD) are combined with 3 biological replicates (n=15 embryos/group). Statistical significance was determined by two-way ANOVA with Tukey's multiple comparison method as a post-hoc test. (G, H, I) Experimental scheme and representative image of macrophages and neutrophils in zebrafish larvae (*tirap^+/+^* and *tirap^-/-^*). Scale bar: 50 µm. (J, K) Quantification of neutrophils and macrophages number in *tirap* mutant and wild-type zebrafish larvae. Number of neutrophils and macrophages in zebrafish larvae under unchallenged conditions. The number of neutrophils for *tirap^+/+^* (n=33) and *tirap^-/-^* (n=27) and the number of macrophages for *tirap^+/+^* (n=36) and *tirap^-/-^* (n=28) are based on two independent experiments. Statistical significant difference was determined by unpaired t-test, ns, non-significant.

The *tirap* mutant was generated by a point mutation from thymine (T) to adenine (A) that leads to a stop codon located in its TIR domain, resulting in a truncated protein (Figure 1A). This condition resulted in mutant zebrafish lacking the intact Tirap protein, thereby preventing the dimerization with Myd88 in the signaling cascade^19^. The genotype proportion from heterozygote cross revealed that this mutation is non-lethal, as the ratio of genotype was the expected 1:2:1 (Figure 1B). The development of *tirap* mutant zebrafish larvae was further investigated. The results revealed no significant difference in terms of the whole body length of *tirap* mutant larvae at 5 dpf compared to their wild type controls (Figure 1C, D). It is expected that *tirap* mutation in zebrafish embryos affects their ability to respond to microbial PAMPs. To confirm this hypothesis, we injected LPS into the blood island of *tirap^+/+^* and *tirap^-/-^* embryos at 27 hpf^24^. Subsequently, we performed a qRT-PCR experiment to determine the expression of *il1b* and *fosl1a* in the 1-hpi *tirap^+/+^* and *tirap^-/-^* embryos. The *il1b* gene encodes a typical pro-inflammatory cytokine in the downstream TLR signaling cascade during the stimulation by LPS^24^. In a previous study, we have shown that *fosl1a* is specifically regulated by Tlr2 signaling^26^. We found that both *il1b* and *fosl1a* can be significantly induced in *tirap^+/+^* embryos upon LPS infection, while no different expression was found in *tirap^-/-^* embryos compared to mock injection control (Figure 1E, F). The results show that the recognition of LPS in zebrafish embryos requires *tirap,* which is consistent with a previous study in a *myd88* mutant^24^. The results confirm that the *tirap^-/-^* embryos generated in this study have lost the function of Tirap. To investigate whether the *tirap* mutation affects the number of leukocytes, the transgenic lines *tirap*^+/+^ *Tg* (*mpeg1*: *mCherry-F*), *tirap*^+/+^ *Tg* (*mpx: EGFP*), *tirap*^-/-^ *Tg* (*mpeg1: mCherry-F*) and *tirap*^-/-^ *Tg* (*mpx: EGFP*) were imaged at 3 dpf to count the number of neutrophils and macrophages in their tail region under unchallenged condition (Figure 1G, H, I). The counting results found no significant difference in the number of neutrophils (Figure 1J) and macrophages (Figure 1K) in *tirap* mutants compared to their wild type controls under unchallenged condition.

### 3.2. Transcriptomic profiling of tirap and myd88 mutant zebrafish

**Figure 2:**
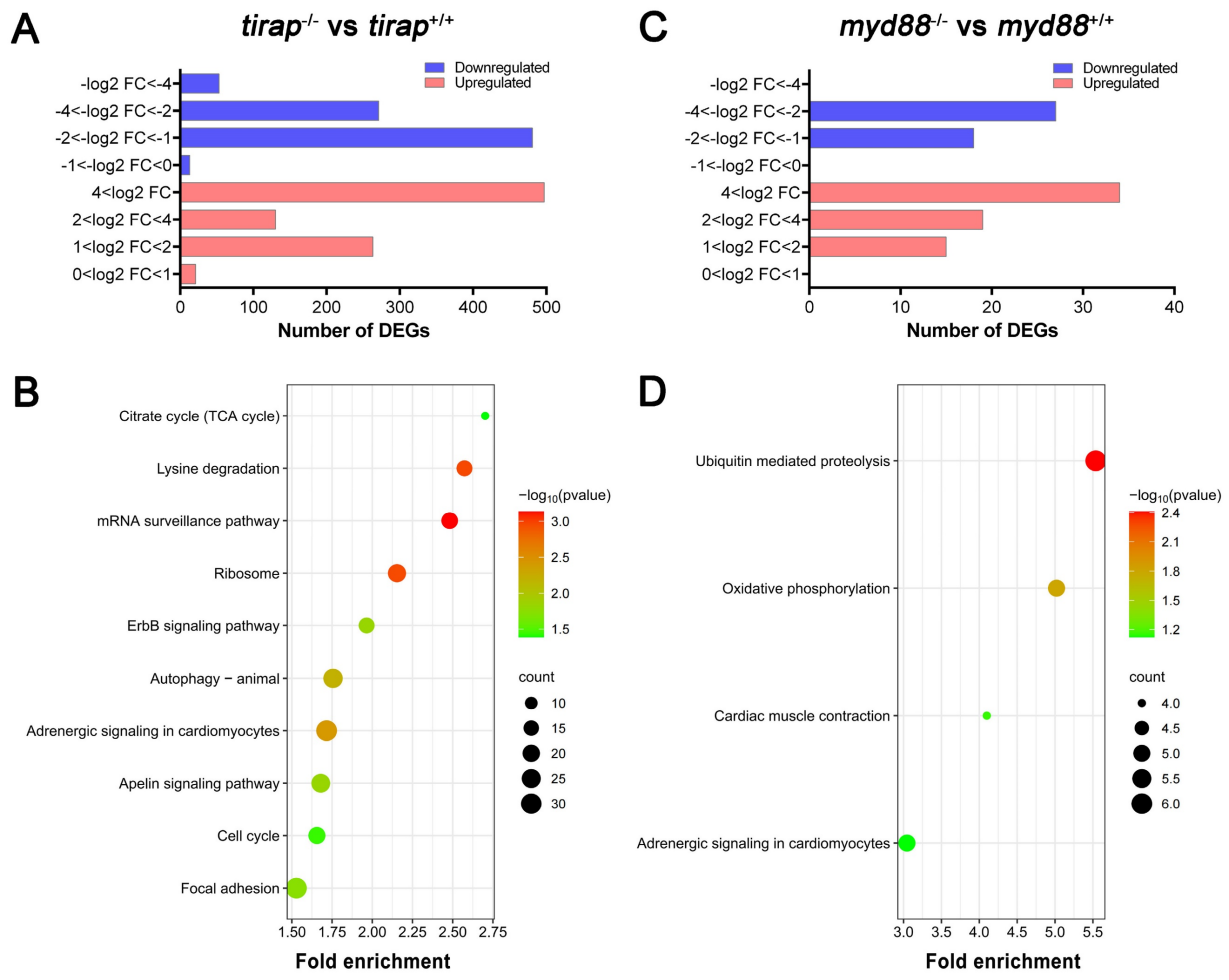
Transcriptomic profiles of *tirap* mutant and *myd88* mutant zebrafish larvae measured by RNAseq. (A) Overview of the distribution of differentially expressed genes (DEGs) log2 fold change in *tirap*^-/-^ versus *tirap*^+/+^ zebrafish larvae. (B) Overview of the distribution of DEGs log2 fold change in *myd88*^-/-^ versus *myd88*^+/+^ zebrafish larvae. DEGs were assessed by padj value less than 0.05. Down-regulated gene sets are shown in blue, and up-regulated gene sets are shown in red. (C) KEGG pathway enrichment analysis of *tirap*^-/-^ versus* tirap*^+/+^. (D) KEGG pathway enrichment analysis of *myd88*^-/-^ versus *myd88*^+/+^. Significantly enriched KEGG pathway terms for DEGs of *tirap*^-/-^ versus *tirap*^+/+^ groups were determined by using the hypergeometric test/Fisher's exact test, with a threshold of *p* value < 0.05, which were adjusted using the Benjamini and Hochbery FDR correction. For *myd88*^-/-^ versus *myd88*^+/+^ group, significantly enriched KEGG pathway terms for DEGs were determined by using the hypergeometric test/Fisher's exact test, with a threshold of *p* value < 0.1.

In order to compare systemic effects of the knock out of the *tirap* and *myd88* genes on transcriptional pathway regulation, we performed whole-larval level transcriptomic studies on mutants and their corresponding sibling wild type controls under unchallenged conditions. For the *tirap* mutant, the number of significantly differentially expressed genes (DEGs) based on the padj value less than 0.05 in the *tirap* mutants compared to the wild type controls was 1729, including 911 upregulated genes and 818 downregulated genes (Figure 2A). Then for comparing the transcriptional characteristics between the *tirap* mutant and *myd88* mutant, we also performed RNAseq of *myd88* mutant zebrafish larvae and their corresponding wild type controls. 113 DEGs were found in *myd88* mutant larvae, including 68 upregulated genes and 45 downregulated genes, compared with the wild type controls based on the padj value less than 0.05 (Figure 2B). For gene enrichment analysis of DEGs, KEGG pathway enrichment analysis was performed using the DAVID bioinformatics program (http://david.abcc.ncifcrf.gov/). In *tirap* mutant larvae, significantly enriched KEGG pathways include citrate cycle (TCA cycle), lysine degradation, mRNA surveillance pathway, ribosome, ErbB signaling pathway, autophagy, adrenergic signaling in cardiomyocytes, apelin signaling pathway, cell cycle and focal adhesion (Figure 2C). KEGG pathways that are significantly enriched in *myd88* mutant larvae include ubiquitin mediated proteolysis, oxidative phosphorylation, cardiac muscle contraction and adrenergic signaling in cardiomyocytes (Figure 2D). The gene annotation of these pathways indicated a common link to various calcium signaling pathways that underlie the changes in the adrenergic signaling and cardiac muscle contraction.

**Figure 3:**
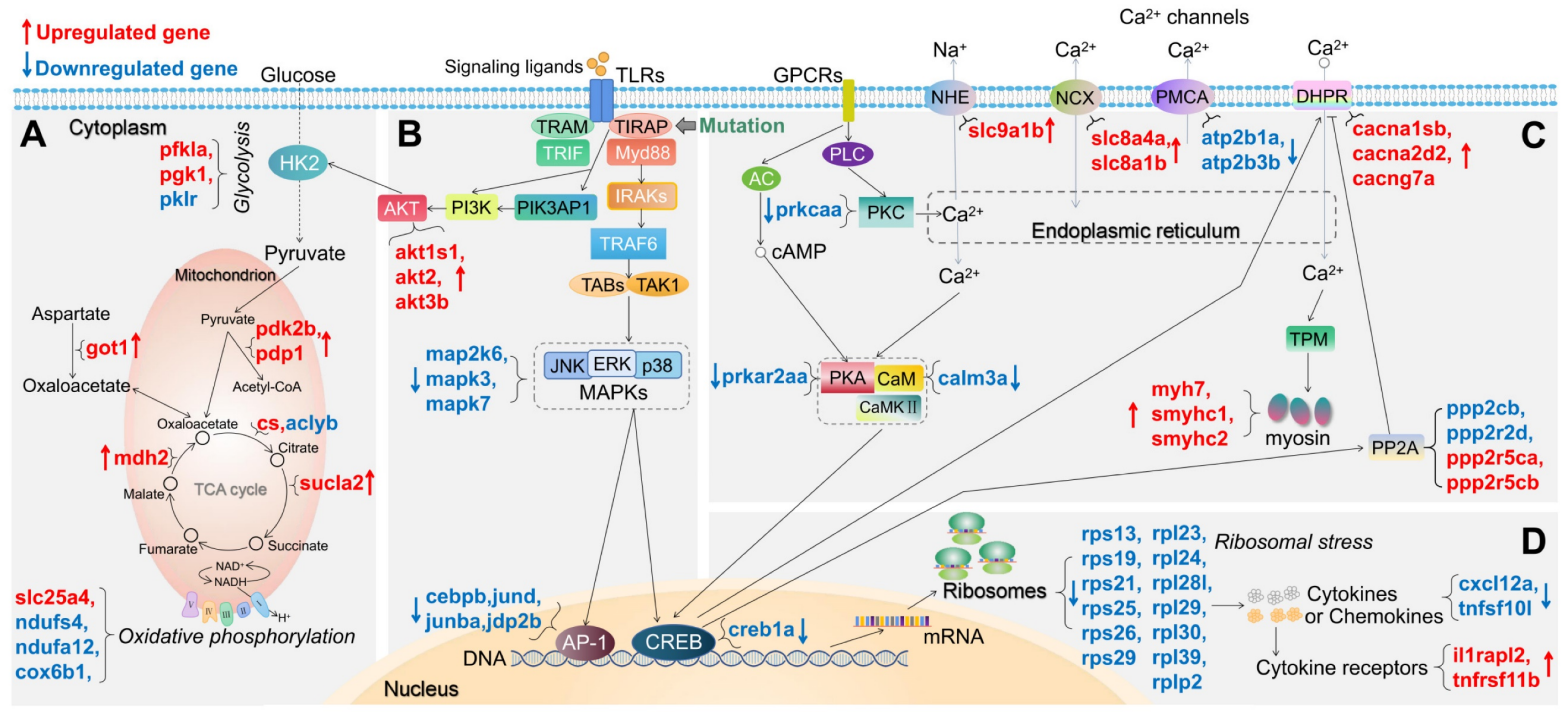
Schematic diagram of significantly differentially expressed genes (DEGs) in the representative pathways in *tirap* mutant zebrafish larvae compared to the wild type controls. (A) Glycolysis and gluconeogenesis, TCA cycle and oxidative phosphorylation related pathways. (B) TLR-associated signaling pathways. (C) Calcium regulation-related pathways. (D) Ribosome-associated pathway. The significance was established as padj value less than 0.05. Red arrows represent significantly upregulated DEGs, blue arrows represent significantly downregulated DEGs. The link between TIRAP and PIK3AP1 refers to Luo, *et al.*^59^. The link between CREB and DHPR refers to Antony, *et al.*^44^. The link between CREB and PP2A refers to Chen, *et al.*^60^. Other links refer to KEGG Pathway Database (https://www.genome.jp/kegg/pathway.html).

**Figure 4:**
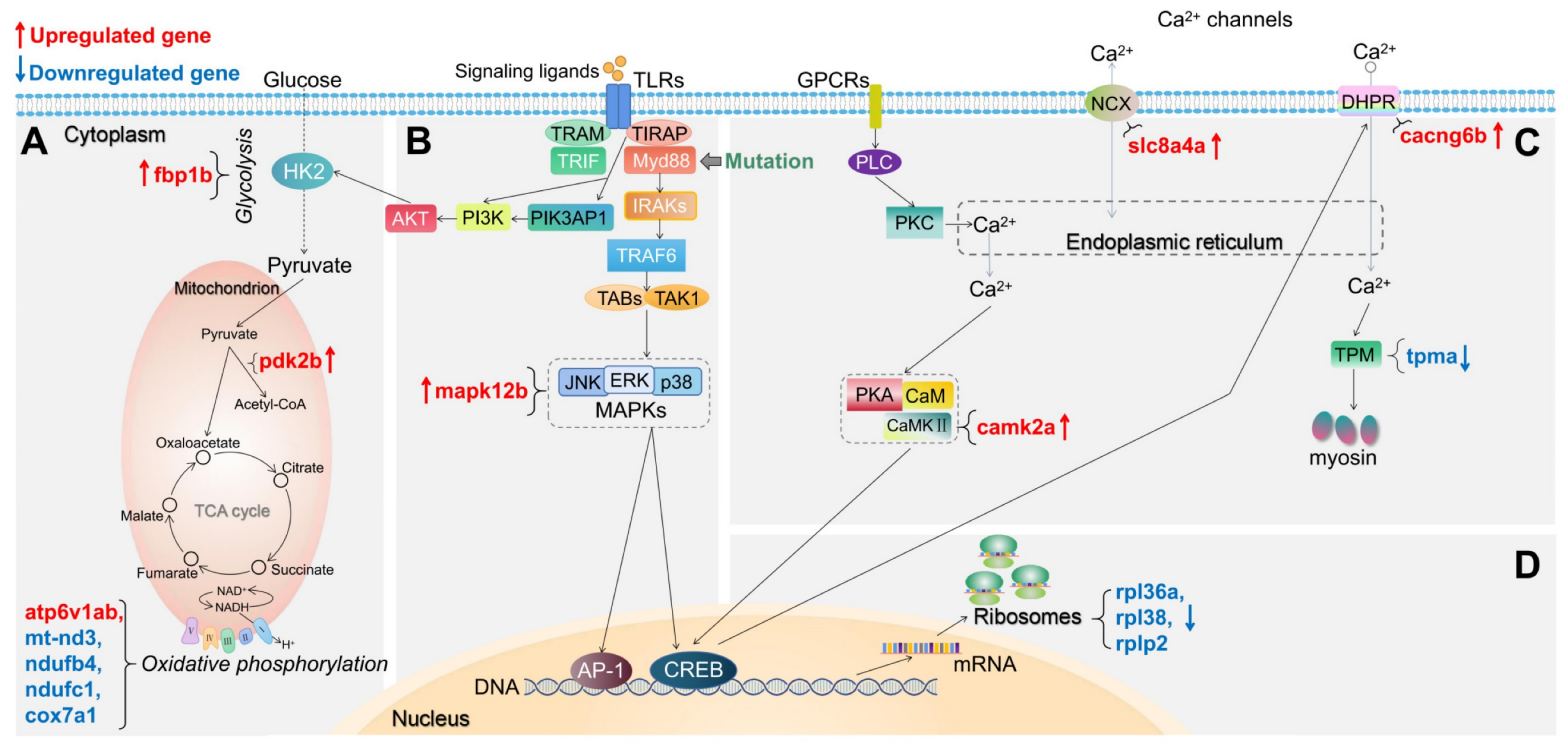
Schematic diagram of significantly differentially expressed genes (DEGs) in the representative pathways in myd88 mutant zebrafish larvae compared to the wild type controls. (A) Glycolysis and gluconeogenesis, TCA cycle and oxidative phosphorylation related pathways. (B) TLR-associated signaling pathways. (C) Calcium regulation-related pathways. (D) Ribosome-associated pathway. The significance was established as padj value less than 0.05. Red arrows represent significantly upregulated DEGs, blue arrows represent significantly downregulated DEGs. The link between TIRAP and PIK3AP1 refers to Luo, *et al.*^59^. The link between CREB and DHPR refers to Antony, *et al.*^44^. Other links refer to KEGG Pathway Database (https://www.genome.jp/kegg/pathway.html).

For both *tirap* mutant and *myd88* mutant, we summarized the changes in expression of DEGs in a number of pathways associated with TLR and calcium signaling and the TCA cycle in Figures 3 and 4, respectively. For example, in glycolysis and gluconeogenesis and TCA cycle pathway, in the *tirap* mutant, *pfkla*, *pgk1*, *got1*, *pdk2b*, *pdp1*, *cs*, *mdh2* and *sucla2* are upregulated, while only *pklr* and *aclyb* are downregulated (Figure 3A). In oxidative phosphorylation pathway, only *slc25a4* is upregulated while *ndufs4*, *ndufa12* and *cox6b1* are downregulated in the *tirap* mutant (Figure 3A). In addition, in the TLR-associated signaling and cytokine production pathway, *akt1s1*, *akt2*, *akt3b*, *il1rapl2* and *tnfrsf11b* are upregulated. In contrast, *map2k6*, *mapk3*, *mapk7*, *cebpb*, *jund*, *junba*, *jdp2b*, *nkap*, *cxcl12a*, *c1qtnf12* and *tnfsf10l* are downregulated in the *tirap* mutant compared with the wild type controls (Figure 3B,D). In calcium regulation-related pathways, *cacna1sb*, *cacna2d2b*, *cacng7a*, *ppp2r5ca*, *ppp2r5cb*, *slc8a1b*, *slc8a4a*, *slc9a1b*, *myh7*, *smyhc1* and *smyhc2* are upregulated, while *ppp2cb*, *ppp2r2d*, *atp2b1a*, *atp2b3b*, *calm3a*, *prkcaa* and *creb1a* are downregulated in the *tirap* mutant (Figure 3C). In addition, we see a large group of ribosomal genes that are downregulated (*rps13*, *rps19*, *rps21*, *rps25*, *rps26*, *rps29*, *rpl23*, *rpl24*, *rpl28l*, *rpl29*, *rpl30*, *rpl39* and *rplp2*) in the *tirap* mutant (Figure 3D). The details of these DEGs from the representative pathways in *tirap*^-/-^ versus *tirap*^+/+^ zebrafish larvae groups are shown in Supplementary Table 2.

For the *myd88* mutant, we also summarized the changes in expression of DEGs in representative pathways associated with TLR signaling in Figure 4. In glycolysis and gluconeogenesis and TCA cycle pathway, in the *myd88* mutant, *fbp1b* and *pdk2b* are upregulated (Figure 4A). In oxidative phosphorylation pathway, only *atp6v1ab* is upregulated while *mt-nd3*,* ndufb4*,* ndufc1* and* cox7a1* are downregulated in the *myd88* mutant (Figure 4A). In the TLR-associated signaling and cytokine production pathway, only *mapk12b* is upregulated in the *myd88* mutant compared with wild type controls (Figure 4B). In calcium regulation-related pathways, *slc8a4a*,* camk2a* and* cacng6b* are upregulated, while only *tpma* is downregulated in the *myd88* mutant (Figure 4C). Ribosomal genes that are downregulated are *rpl36a*,* rpl38* and *rplp2* in the *myd88* mutant (Figure 4D). The details of these DEGs from the representative pathways in *myd88*^-/-^ versus *myd88*^+/+^ zebrafish larvae groups are shown in Supplementary Table 3.

In conclusion, the effects of knocking out the *tirap* and *myd88* genes are very different both quantitatively and in the affected pathways as discussed below. Most notably, there are significant differences in pathways related to metabolism.

### 3.3. Metabolomic profiling of tirap and myd88 mutant zebrafish

**Figure 5:**
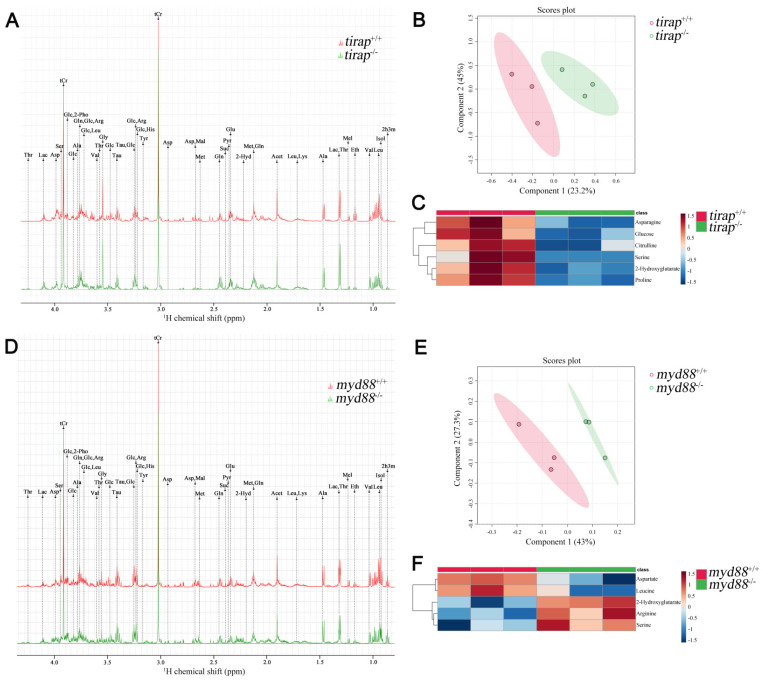
Metabolic profiles of *tirap* mutant and *myd88* mutant zebrafish larvae measured by ^1^H NMR and analysis of PLS-DA and heat map. (A) The representative one-dimensional ^1^H NMR spectra of *tirap* wild type and mutant zebrafish larvae measured by NMR spectrometry. Spectra from chemical shift 0.8 to 4.3 were assigned to specific metabolites. (B) PLS-DA analysis of *tirap* wild type and mutant groups. n = 3, each replicate represents 120 pooled larvae. (C) Heat map analysis of significantly altered metabolites detected in *tirap* mutants (*P* < 0.05). n = 3, each replicate represents 120 pooled larvae. (D) The representative one-dimensional ^1^H NMR spectra of *myd88* wild type and mutant zebrafish larvae measured by NMR spectrometry. Spectra from chemical shift 0.8 to 4.3 were assigned to specific metabolites. (E) PLS-DA analysis of *myd88* wild type and mutant groups. n = 3, each replicate represents 120 pooled larvae. (F) Heat map analysis of significantly altered metabolites detected in *myd88* mutants (*P* < 0.05). Thr threonine, Lac lactate, Asp aspartate, Ser Serine, tCr total creatine (creatine + phosphocreatine), Glc Glucose, 2-Pho 2-Phosphoglycerate, Ala alanine, Gln glutamine, Arg arginine, Leu leucine, Val valine, Gly glycine, Tau taurine, His Histidine, Tyr tyrosine, Mal Malate, Met Methionine, Suc Succinate, Pyr Pyruvate, Glu glutamate, Acet Acetate, Lys lysine, Mel Melatonin, Eth Ethanol, Isol Isoleucine, 2h3m 2-Hydroxy-3-methylvalerate, 2-Hyd 2-Hydroxyglutarate.

The *tirap* mutation caused greater differences at the transcriptional level compared to its wild type control, as evidenced by a much higher number of DEGs than the *myd88* mutation under unchallenged conditions. To compare the effects of mutations in *tirap* and *myd88* on a broader level, we used 1-D ^1^H NMR spectroscopy to investigate the differences in metabolite levels between *tirap* mutant and wild type zebrafish larvae at 5 dpf, and also between *myd88* mutant and wild type control at 5 dpf. Representative 1-D 1H NMR spectra of *tirap* mutant and wild type groups are shown in Figure 5A. The chemical shifts of the 1-D ^1^H NMR spectra were assigned according to the chemical shifts of the reference metabolites from the Chenomx 600 MHz library (version 11). Subsequently the 1-D ^1^H-NMR spectra of *tirap* wild type and mutant groups were examined by multivariate analysis using the Partial least squares-discriminant analysis (PLS-DA) modeling to investigate whether these two experimental groups can be well discriminated. PLS-DA results showed clustering of *tirap* wild-type and mutant groups. This suggests that *tirap* deficiency in zebrafish larvae leads to metabolic changes (Figure 5B). For the *tirap* mutants, six significantly downregulated metabolites including proline, 2-Hydroxyglutarate, asparagine, glucose, serine and citrulline were detected compared to wild-type controls (Figure 5C). The details of these significantly altered metabolites in *tirap*^-/-^ versus *tirap*^+/+^ zebrafish larvae groups are shown in Supplementary Table 4.

Similarly, representative 1-D ^1^H NMR spectra of *myd88* mutant and wild type groups are shown in Figure 5D. PLS-DA results of *myd88* mutants showed clustering of *myd88* wild-type and mutant groups (Figure 5E). Five significantly altered metabolites were detected in the *myd88* mutants compared to wild-type controls, with aspartate and leucine being downregulated and serine, arginine and 2-Hydroxyglutarate being upregulated (Figure 5F). The details of these significantly altered metabolites in *myd88*^-/-^ versus *myd88*^+/+^ zebrafish larvae groups are shown in Supplementary Table 5.

### 3.4. tirap mutant larvae have higher bacterial burden after M. marinum infection

**Figure 6:**
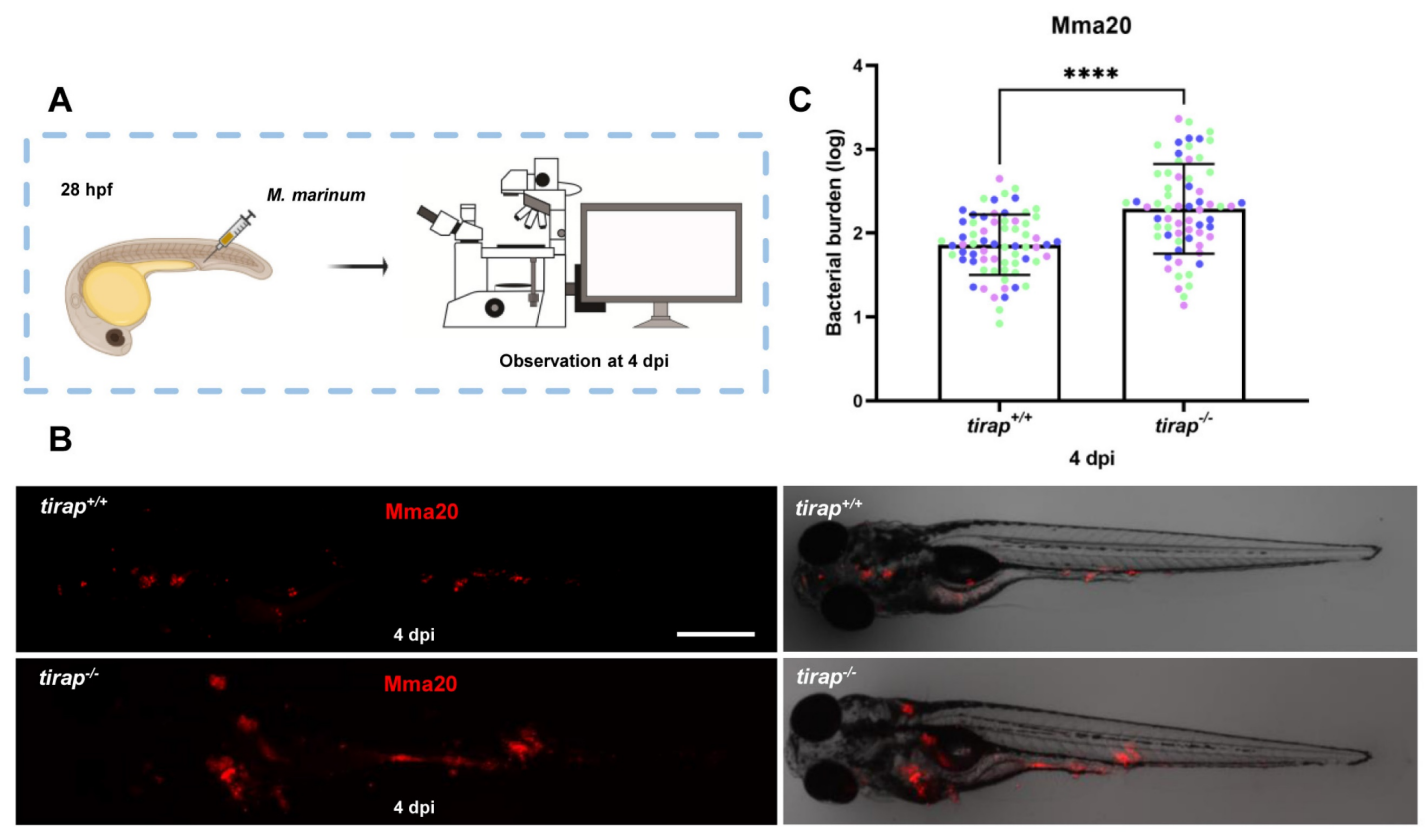
Quantification of bacterial burden after *M. marinum* infection in *tirap* mutant and wild type zebrafish larvae. (A) *tirap*^+/+^ and *tirap*^-/-^ zebrafish were infected with mCherry-labeled *M. marinum* strain Mma20 by caudal vein injection at 28 hours post fertilization (hpf). (B) Representative images were taken after 4 days of infection (dpi), respectively. (C) Bacterial burdens at 4 dpi were quantified using bacterial fluorescence pixels. Data were combined from 3 independent experiments, for *tirap*^+/+^, n=67, for *tirap*^-/-^, n=65. ****, *P* <0.0001. Scale bar: 500 µm.

To explore the effect of *tirap* on immune response after mycobacterial infection, the strain Mma20 was injected into the blood island of *tirap* mutant larvae and their wild type controls at 28 hpf. Quantitative results after imaging at 4 dpi showed that *tirap* mutant larvae have significantly higher bacterial burden compared to their wild type controls (Figure 6).

### 3.5. Tirap mediates neutrophil recruitment to wounds, but not macrophages

**Figure 7:**
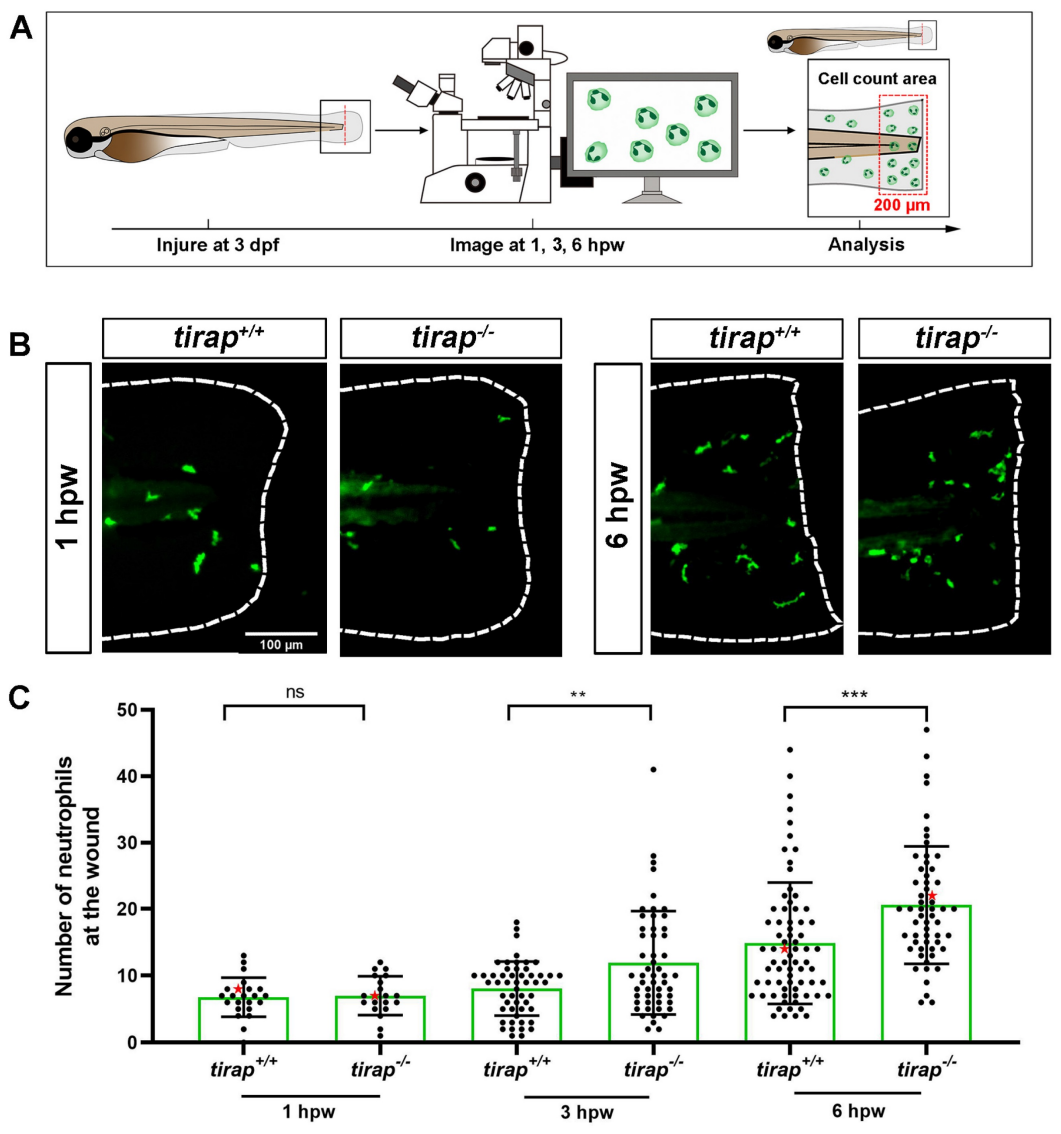
The number of recruited neutrophils upon tail wounding in zebrafish larvae. (A) Schematic representation of experiment. The wounded zebrafish larvae were imaged at designated times 1 hour post wounding (hpw), 3 hpw and 6 hpw. (B) Representative images of *tirap^+/+^* and *tirap^-/-^* at 1 hpw and 6 hpw. Scale bar: 100 µm. (C) Number of neutrophils recruited to tail region upon tail wounding at 1, 3, 6 hpw. Red stars indicate the representative images shown in the panel B. The number of neutrophils at 1 hpw from one independent experiment for *tirap^+/+^* (n=23) and *tirap^-/-^* (n=20), at 3 hpw for *tirap^+/+^* (n= 52) and *tirap^-/-^* (n= 53) and at 6 hpw for *tirap^+/+^* (n=72) and *tirap^-/-^* (n=57) are based on three independent experiments. Statistically significant difference was determined by nonparametric tests: Mann-Whitney and Kolmogorov-Smirnov, ns, non-significant; **, *P* < 0.01, ***, *P* < 0.001.

After observing no difference in neutrophil numbers between *tirap* mutant larvae and their wild type controls under the unchallenged condition (Figure 1), the tail wounding model was next used to investigate whether *tirap* had an effect in inflammation resolution. In 3 dpf zebrafish larvae, after tail wounding, the number of recruited neutrophils to the wounded area (in a range close to 200 µm from the tail wound edge) was counted at 1, 3 and 6 hpw (Figure 7A). The results showed no difference in the number of neutrophils recruited between *tirap* mutant and wild type larvae at 1 hpw. However, at 3 hpw and 6 hpw, *tirap* mutant larvae recruited significantly more neutrophils than wild type controls, indicating that *tirap* is involved in regulating neutrophil recruitment in inflammatory resolution (Figure 7B-C). For macrophage recruitment at the wounding part, there was no significant difference in the number of macrophages recruited between *tirap* mutant and wild type larvae at 1, 2, 4 and 6 hpw, suggesting that *tirap* has no effect on macrophage recruitment after wounding (Supplementary Figure 1).

### 3.6. Tirap affects neutrophil migration speed but not directional persistence upon tail wounding

**Figure 8:**
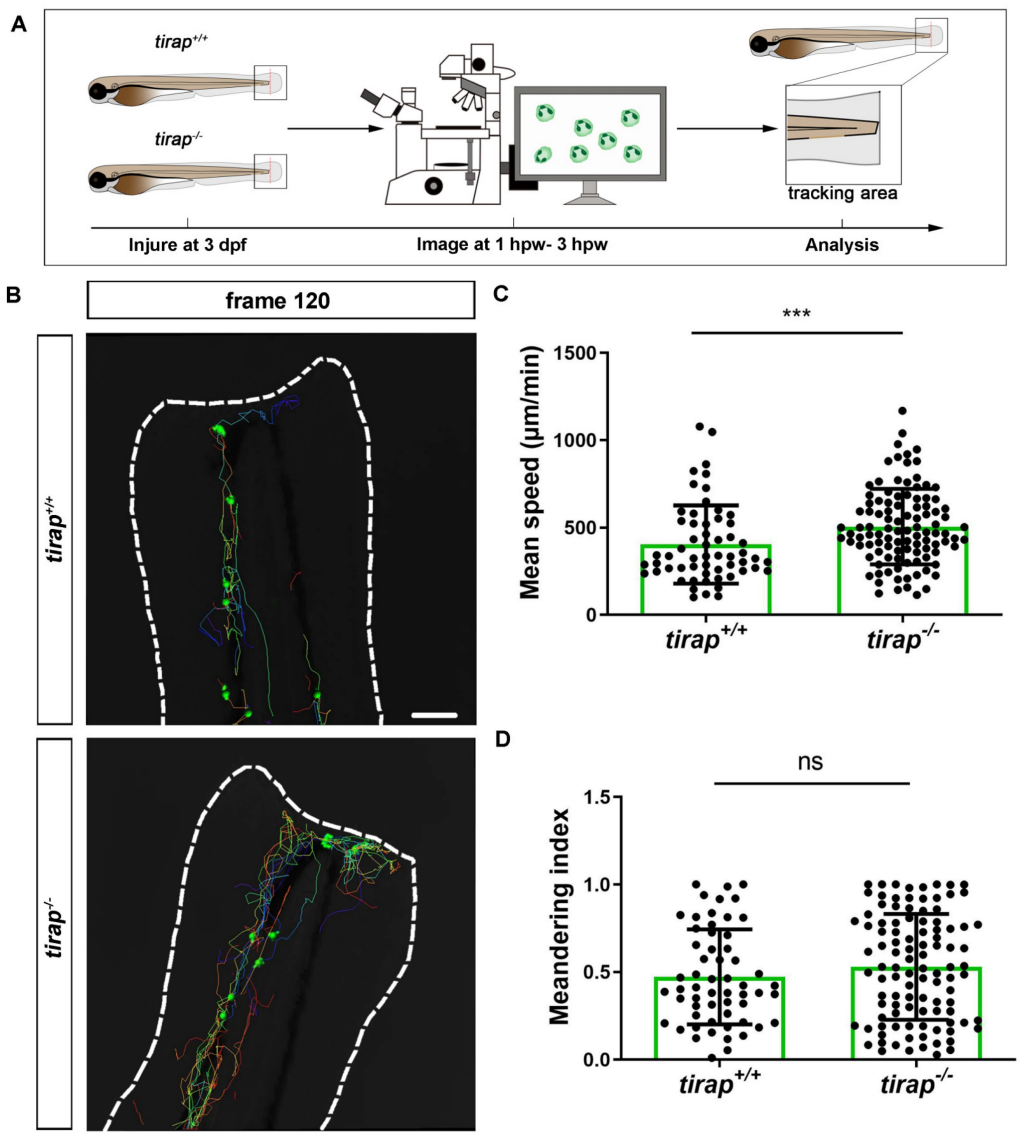
Quantification of neutrophil behaviour upon tail wounding in zebrafish larvae. (A) Schematic representation of experiment. (B) Representative image of neutrophil migration at frame 120 (3 hpw) (C-D) Quantification of neutrophil migratory capability of 3 dpf *tirap* larvae upon tail wounding. Mean speed (C) and meandering index (D) were quantified using IMARIS Software. The mean speed of neutrophils is measured as total displacement of neutrophils divided by the number of frames. Data consists of 3 *tirap^+/+^* and 3 *tirap^-/-^* larvae. Statistically significant difference was determined by unpaired t test with Welch's correction, ns, non-significant; ***, *P* < 0.001.

Having established that *tirap* can regulate neutrophil recruitment after wounding, we further investigated neutrophil behaviour in *tirap* mutant larvae, such as neutrophil migration speed and directional persistence. Firstly, we investigated the migratory behaviour of neutrophils from *tirap* mutant larvae under unchallenged condition. Therefore, live imaging of the caudal haematopoietic tissue (CHT) region of zebrafish larvae was performed. The neutrophil mean speed and meandering index were specified as two indicators to assess the migratory behaviour of neutrophils. Mean speed is obtained by dividing the total displacement by the travel time. Meandering index corresponds to the neutrophil distance between first and the last frame (net displacement) divided by their total distance (total displacement) and can be used to indicate the direction of migration^10^. The results revealed no significant differences between *tirap* mutant and wild type larvae in terms of neutrophil migration speed and direction under unchallenged conditions (Supplementary Figure 2). Subsequently, the role of *tirap* in neutrophil motility behaviour upon tail wounding was investigated. Live imaging of the caudal region of wounded zebrafish larvae was performed from 1 hpw to 3 hpw for 2 hours in total (Figure 8A-B). In terms of migration speed, the results showed that the mean speed of neutrophils in the *tirap* mutant larvae was significantly faster compared to the wild type controls (Figure 8C). As for the direction of migration, the results showed no significant difference in the meandering index of neutrophils in the *tirap* mutant larvae compared to the wild type (Figure 8D). These results suggest that *tirap* deficiency affects the speed of neutrophil migration at the caudal wound area, but not the direction of migration.

### 3.7. Gene expression profiles of immune-related genes upon tail wounding

**Figure 9:**
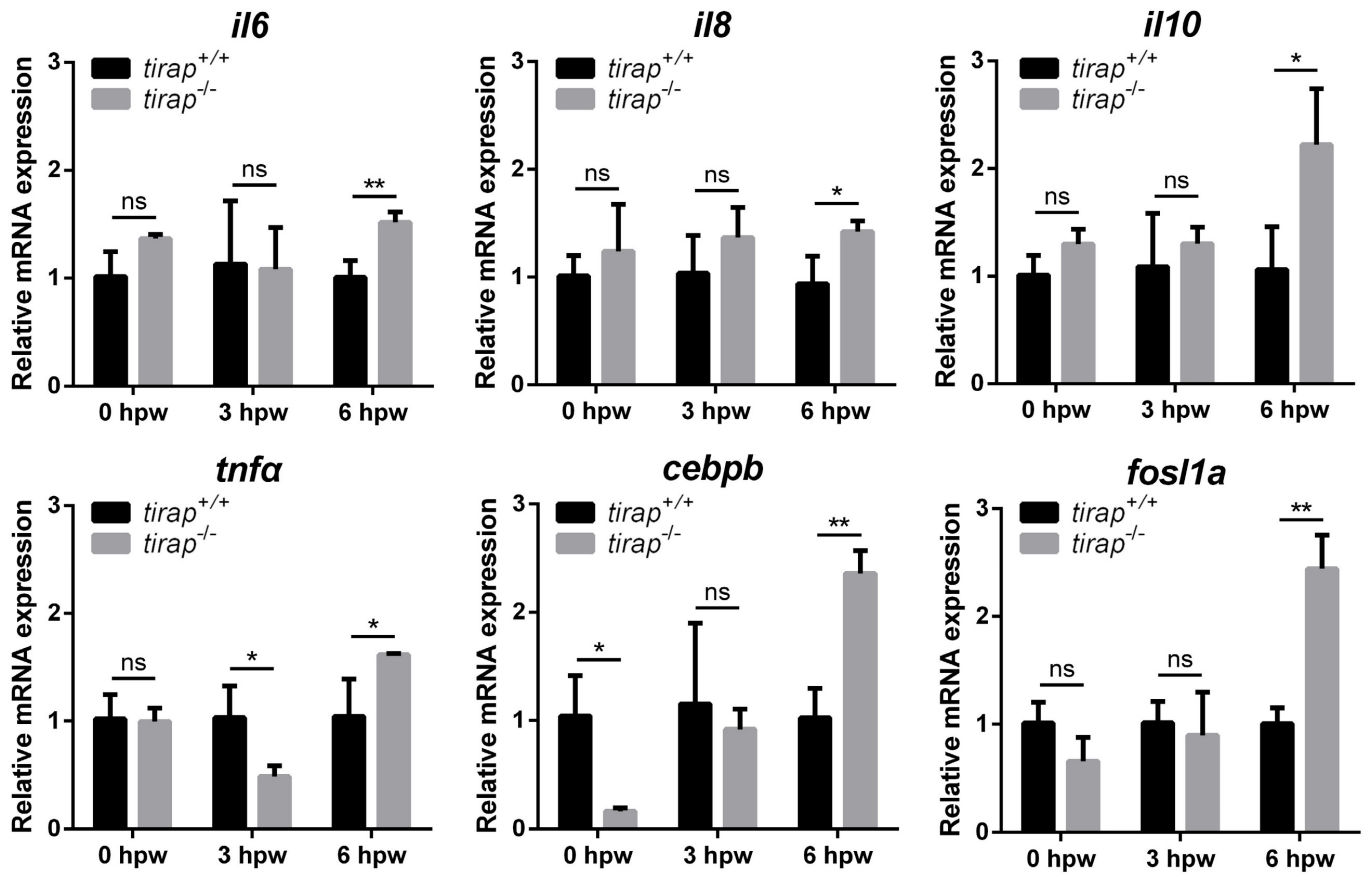
Relative expression of immune-related genes upon tail wounding in *tirap* mutant zebrafish larvae. hpw, hour post wounding; ns, non-significant; *, *P* < 0.05; **, *P* < 0.01.

To further investigate the molecular mechanisms by which tirap affects post-injury neutrophil migration, the relative expression of downstream genes associated with TLR signaling was examined in zebrafish larvae after tail wounding. These genes include il6, il8, il10, tnfa, cebpb and fosl1a. The results showed no significant difference in the relative expression of the genes examined in the tirap mutant versus wild type controls before tail wounding, except for cebpb, which was expressed lower in the tirap mutant than in wild types. At 3 hours post wounding (hpw), with the exception of tnfa, there was no significant difference in the relative expression of other examined genes in the tirap mutants and wild type controls. However, at 6 hpw, all tested genes showed significantly elevated relative expression in the tirap mutant larvae compared to the wild type controls (Figure 9).

## 4. Discussion

In this study, we have investigated the function of *tirap* and *myd88* in transcriptomic and metabolomic regulation under unchallenged conditions by obtaining RNAseq and NMR profiles in mutant zebrafish larvae and their wild type controls. We made further comparisons with our previously published *tlr2* mutant data^23^. In Table 1, we systematically summarize the transcriptomic and metabolomic characteristics of three mutants, *tirap*, *myd88* and *tlr2,* as compared to the three sibling control lines under unchallenged conditions. In terms of transcriptomic characteristics, the number of DEGs in *tirap*^-/-^ versus *tirap*^+/+^ group is much higher than in the *myd88*^-/-^ versus *myd88*^+/+^ group and in the *tlr2*^-/-^ versus *tlr2*^+/+^ group. No overlapping DEGs were found in the three mutants (Supplementary Figure 3). Also, among the DEGs, there are more genes involved in TLR signaling-related pathways in *tirap*^-/-^ versus *tirap*^+/+^ group than in the other two mutants (Table 1A). For example, *akt1s1*, *akt2* and *akt3b* are only upregulated in *tirap* mutant larvae. Interestingly, it was shown previously that TIRAP and MyD88 are both required for TLR2/6-mediated PI3K-dependent mouse macrophage membrane ruffling at the leading edge and PIP3 formation, whereas only TIRAP is essential for Akt phosphorylation^34^. Therefore, a specialized function of TIRAP is also apparent in mammalian cells.

In addition, the *tirap* mutant is the only one of the three mutants that causes numerous down-regulations in the expression of transcripts of ribosomal proteins. Since ribosomal protein genes are under cotranslational regulation upon ribosomal stress^35^, the cohort of down-regulated ribosomal protein genes might represent ribosomal stress. It has been shown that down regulation of ribosomal proteins such as RPS19 leads to ribosomal stress and severe proteomic changes for instance in proteins related to cytoskeletal reorganisation^36^. Previous studies have also shown that ribosomal proteins have functions in resolving physiological inflammation. For instance, ribosomal protein L13a depletion abrogates the endogenous translation control of several chemokines in macrophages^37^. Furthermore, ribosomal protein L13a-dependent translational silencing suppresses the synthesis of a series of inflammatory proteins in monocytes and macrophages^38^. In our study, we also found some downregulated chemokine (*cxcl12a*) and cytokine (*tnfsf10l*) genes and upregulated cytokine receptor genes (*il1rapl2* and *tnfrsf11b*) in the *tirap* mutant. This suggests that Tirap is associated with the regulation of the inflammatory response, which might be the result of altered expression of ribosomal proteins.

The down-regulation of inflammatory cytokine and chemokine expression observed in the *tirap* mutant larvae may also be attributed the result of a defect in p38 mitogen-activated protein kinase (p38 MAPK) signaling. The downstream p38 MAPK signaling axis of TLR signaling controls AP-1-mediated proinflammatory cytokines expression^39^, ^40^. In the *tirap* mutant, *map2k6*, *mapk3* and *mapk7* were downregulated and AP-1 transcription factors, sush as *cebpb*, *jund*, *junba* and *jdp2b*, were also downregulated. However, in *myd88* and *tlr2* mutants, no changes in the expression of AP-1-related genes were detected. Notably, previous studies have shown that TIRAP brings PKCδ and p38 in close proximity to each other to form a complex and this complex is required for AP-1 mediated inflammatory responses^41^. Further studies are needed to identify a specific function of TIRAP also in mammalian inflammation responses related to AP-1 activation.

Except for AP-1, TLRs can also trigger activation of other transcription factors, such as cAMP responsive element binding protein (CREB)^42^. Our results show that the *creb1a* gene is only downregulated in the *tirap* mutant larvae. Interestingly, TIRAP was shown in a previous study to function as a key upstream regulator of CREB to control both pro- and anti-inflammatory gene expression^43^. In addition, it was found that CREB mediated CACNA1S expression in the context of *Mycobacterium tuberculosis* (*Mtb*) infection, which is involved in the regulation of calcium homeostasis^44^. In the *tirap* mutant, we found several DEGs related to calcium channels regulation. In contrast only one different calcium channel protein was differentially expressed in the *myd88* mutant whereas no calcium channel genes were differentially expressed in the *tlr2* mutant (Table 1A). This suggests that Tirap has specific roles in maintaining calcium homeostasis.

Although the *tirap* and *myd88* mutations do not affect the growth of zebrafish larvae (Figure 1)^24^, we show that both the *tirap*^-/-^ versus *tirap*^+/+^ group and *myd88*^-/-^ versus *myd88*^+/+^ group showed several differentially expressed genes associated with the glycolysis and gluconeogenesis and TCA cycle. In the *tirap* mutant, we found the up-regulation of the *pfkla* gene encoding a phosphofructokinase and *pgk1* encoding a phosphoglycerate kinase 1 and down-regulation of the gene *pklr* encoding a pyruvate kinase (Figure 3). This suggests that Tirap is involved in regulating the glycolytic pathway. However, in the *myd88* mutant, only the gene *fbp1b* which encodes a fructose-1,6-bisphosphatase was found to be upregulated (Figure 4). In our previous study of the transcriptome of *tlr2* mutant zebrafish larvae, the expression of the *gpib* and *pfkma* genes encoding a glucose-6-phosphate isomerase and phosphofructokinase, respectively, were downregulated^23^. These results indicate different roles of Tirap, Myd88 and Tlr2 in regulating glucose metabolism.

To further explore how the *tirap* mutant and *myd88* mutant affect metabolism, we performed NMR spectroscopy for metabolomic analysis. In the *tirap* mutant, we found a decrease in the contents of proline, asparagine, serine and citrulline, and also a decrease in 2-hydroxyglutarate and glucose (Table 1B). However, the *myd88* mutant was found to have an unaltered glucose level (Table 1B). Combined with the transcriptomic data, we speculate that the reduced glucose in the *tirap* mutant may result from the elevated expression of genes involved in the glycolytic process. In contrast, our published data about *tlr2* mutant zebrafish larvae, showed that higher glucose levels were detected in the *tlr2* mutant, which was associated with lower gene expression in glycolysis pathway^23^. These results suggest that Tirap and Tlr2 control glucose metabolism in a different way under unchallenged conditions.

Our transcriptional and metabolic results illustrate that the alterations triggered by the three mutants are quite different under unchallenged conditions, with the *tirap* mutation leading to a broader range of alterations of genes related the modulation of the inflammatory response. Therefore, we further explored the role of *tirap* in response to *M. marinum* infection. The higher bacterial burden detected in the *tirap* mutant shows a protective role of Tirap against mycobacterial infection. Previous studies have shown that *tlr2* and *myd88* mutations also lead to a higher bacterial burden using the same infection system^26^, ^45^. TLR signaling has been reported to play a role in host defense against mycobacteria, such as *Mtb*^46^, ^47^. However, the function of the *tirap* gene in mycobacterial infection has not been much studied. Surprisingly, in a mouse infection model of tuberculosis it was shown that Tirap heterozygous mouse mutants were more resistant to infection^48^. This negative function of Tirap was linked to a Cish-dependent acidification of macrophages. Considering that the function of Cish is tightly linked with T-cell functions^49^, it is possible that the absence of adaptive immune components in the zebrafish larval model explains the difference with the results of the mouse infection model. However, the protective function of Tirap would not be surprising, considering its function in inflammation and metabolism in the unchallenged conditions. It has been shown that metabolic regulation such as the shift to glycolysis is an important component of host defense in a mouse *Mtb* model^50^. The switch to glycolysis in *Mtb*-exposed cells relies on TLR2 recognition and is partly dependent on the intracellular AKT-mTOR axis^51^. Our metabolomic data show that *tirap* has a regulatory role in glycolytic process, and transcriptomic data show that *tirap* mutation leads to an increased expression of several *akt* genes. It is therefore interesting to further study the function of Tirap during infection and inflammation in more detail in other model systems which also reflect adaptive immune components.

**Figure 10:**
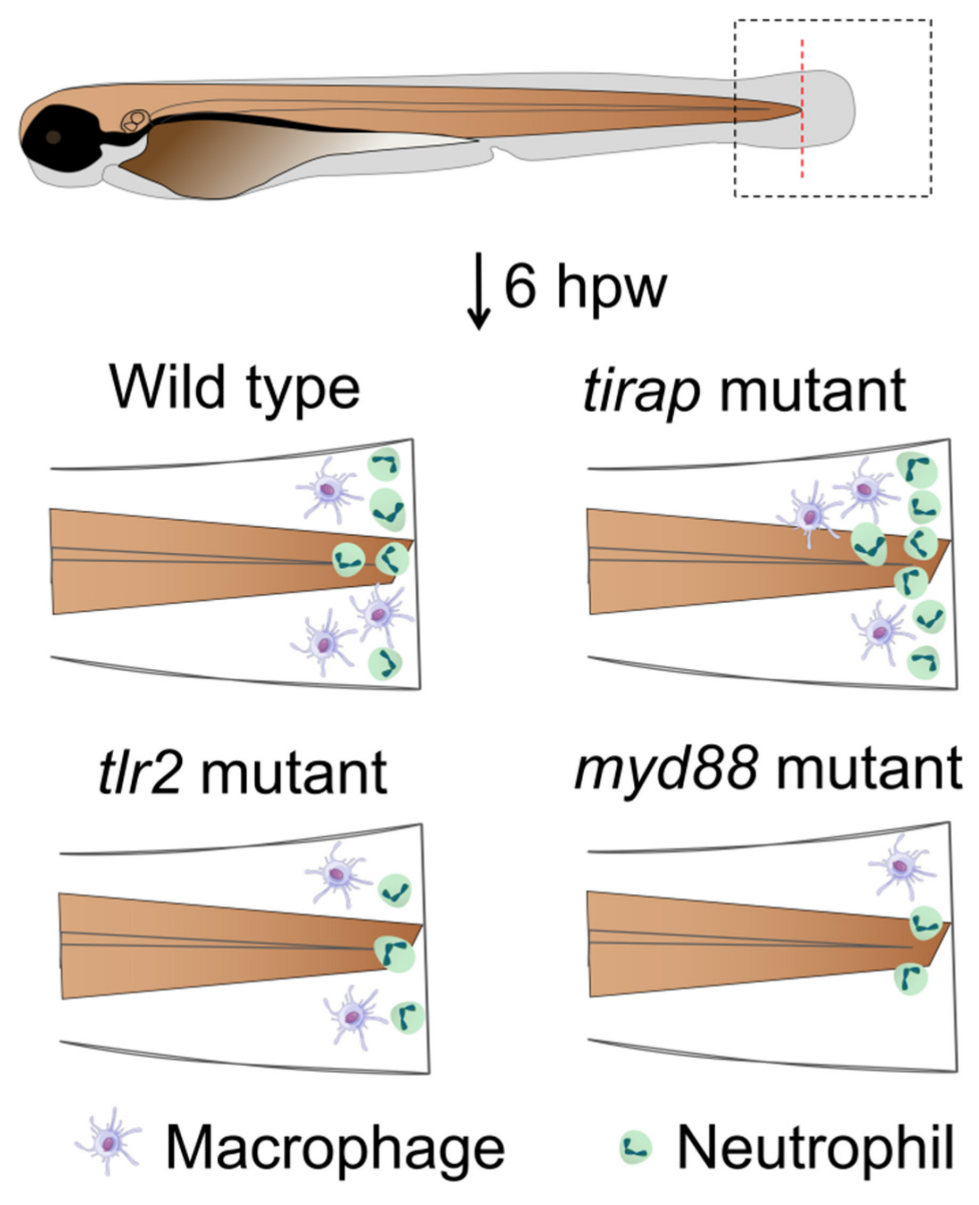
Graphical summary of *tirap* mutant compared to *tlr2* and *myd88* mutants in affecting leukocyte migration behavior upon tail wounding in zebrafish larvae. hpw, hour post wounding.

In this study, we demonstrated that mutation of the *tirap* gene does not affect the number of neutrophils and macrophages *in vivo* under the unchallenged condition (Figure 1). However, live imaging in a zebrafish larval tail wounding model revealed that the *tirap* mutant larvae recruited more neutrophils at the wounds (Figure 7). In our previous study on *tlr2* and *myd88* in TLR signaling affecting leukocyte migration in zebrafish, it was similarly found that* tlr2* and *myd88* mutation could affect the migratory behaviour of leukocytes towards the wounds^11^. However, in the present study we show that *tirap* has a very a different function in the wounding response compared to *tlr2* and *myd88*. When *tirap* was absent, more neutrophils were recruited to the wound compared to the wild type control (Figure 7), whereas both *tlr2* and *myd88* mutant larvae recruited fewer neutrophils compared to their respective wild type controls (Figure 10, Table 1C). We can speculate that the deficiency of *tirap* may have resulted in impaired reverse migration resulting in a higher number of neutrophils at the wounds at later time points. Future studies using transgenic lines in which Tg(mpx:Dendra) reporters are crossed in our mutant will be essential to further study the function of *tirap* in reverse neutrophil migration. Based on our transcriptome and metabolome study, there are several possible explanations why the* tirap* mutant behaves very different than the *myd88* and *tlr2* mutants in wounding.

In the first place, the* tirap* mutant shows an effect on the transcription of many genes involved in calcium-dependent cytoskeletal regulation. In our results, up-regulation of calcium channels and myosin-related gene expression was specifically found in the *tirap* mutant. Calcium ions serve as the main signal, which induces activation of actin-myosin interactions not only in muscle cells but also in many other cell types^52^-^54^. Myosin activity has been shown to be an important component of defense responses in myeloid cells^55^. It has also been shown that reactive oxygen species play a negative role in *Mtb*-mediated CACNA1S expression. Since reactive oxygen species play a major role in cell migration during wounding^56^. The effect of the *tirap* mutation on expression of* cacna1a* might also be relevant in understanding its function in neutrophil migration. Therefore, the specific function of *tirap* in regulation of calcium-dependent myosin function could explain a higher speed of neutrophils migrating to the wounds in the *tirap* mutant.

Another explanation for the very different effect on cell migration in the *tirap* mutant as compared to *myd88* and *tlr2* mutants might be related to its different function in cytokine or chemokine regulation. Considering that the *tirap* mutant is unique in its down-regulation of *cxcl12a*, this relates to the various observations of the function of this chemokine in neutrophil responses to inflammation. Previous study shows increased neutrophil recruitment to wounds in *cxcl12a* mutant zebrafish larvae^57^. Therefore, our observation that the *tirap* mutant recruited more neutrophils to wounds may result from a down-regulation of *cxcl12a*. However, we have no clue yet as why *tirap* has no function in macrophage migration in contrast to the other mutants. We will need single cell analysis methods for further studies to obtain insights in the specific functions of Tirap in various cell types such as macrophages, neutrophils and T-cells.

Considering the specialized functions of *tirap* compared to *myd88*, it might be relevant to further study whether it can be used as a therapeutic target. Recent studies have demonstrated that the intervention in reverse migration may be a therapeutic target to facilitate resolution of neutrophil inflammation^22^, ^58^. Therefore, strategies to enhance* tirap* expression might be useful to combat hyper inflammatory responses. Yet, since there is still a great lack of knowledge of the function of TIRAP in organismal or cellular models, many further experiments are still needed to unravel the mechanism of interactions between *tirap* and its upstream and downstream partners in resolving inflammation.

## 5. Conclusions

From our study of zebrafish larvae with mutations in *tirap* and *myd88*, we conclude that Tirap has specialized roles in signaling, metabolic control and leukocyte migration upon wounding in zebrafish larvae. The transcriptome results indicate a specialized function in calcium homeostasis and myosin regulation under unchallenged conditions. The metabolomic studies show that *tirap*, like *myd88* and *tlr2*, has a function in control of metabolism under unchallenged conditions. However, the metabolic function of *tirap* is very different from *myd88* and *tlr2*. For instance, the* tirap* mutation leads to lower glucose levels, whereas a *tlr2* mutation leads to higher glucose levels at the systems level. The results of the tail-wound model show that the *tirap* mutant larvae recruited significantly more neutrophils to the wounds than the wild type control, in contrast to *myd88* and *tlr2* mutants. This could be related to a specialized function in the regulation of calcium-channel dependent myosin-actin dynamics, or in the specific regulation of chemokine such as *cxcr12a*. The specialized function of* tirap* as an adapter of TLR signaling makes it an extractive target for therapeutic studies aimed at modulating neutrophil inflammatory responses.

## Supplementary Material

Supplementary figures and tables.

## Figures and Tables

**Table 1:** Simplified phenomics comparison of three mutants

A. Transcriptome			*tirap*^-/-^ vs *tirap*^+/+^	*myd88*^-/-^ vs *myd88*^+/+^	*tlr2*^-/-^ vs *tlr2*^+/+^ 23
DEG number (padj value)			1729*	113*	7
DEG number (s value)			1632	155	149*
TLRs-associated signaling and cytokine production		**↑**	*akt1s1*, *akt2*, *akt3b*, *il1rapl2*, *tnfrsf11b*	*mapk12b*	**―**
		**↓**	*map2k6*, *mapk3*, *mapk7*, *cebpb*, *jund*, *junba*, *jdp2b*, *cxcl12a*, *tnfsf10l*	**―**	*map3k4*
Glycolysis and gluconeogenesis		**↑**	*pfkla*, *pgk1*, *got1*	*fbp1b*	**―**
		**↓**	*pklr*	**―**	*gpib*, *pfkma*
TCA cycle		**↑**	*pdk2b*, *pdp1*, *cs*, *mdh2*, *sucla2*	*pdk2b*	**―**
		**↓**	*aclyb*	**―**	*pck2*
Ribosome		**↑**	**―**	**―**	*rpl10*, *rpl13*, *rrp7a*
		**↓**	*rps13*, *rps19*, *rps21*, *rps25*, *rps26*, *rps29*, *rpl23*, *rpl24*, *rpl28l*, *rpl29*, *rpl30*, *rpl39*, *rplp2*	*rpl36a*, *rpl38*, *rplp2*	**―**
Oxidative phosphorylation		**↑**	*slc25a4*	*atp6v1ab*	*ndufa6*, *mt-nd3*, *ndufs4*, *ndufv3*
		**↓**	*ndufs4*, *ndufa12*, *cox6b1*	*mt-nd3*, *ndufb4*, *ndufc1*, *cox7a1*	**―**
Calcium regulation		**↑**	*cacna1sb*, *cacna2d2b*, *cacng7a*, *ppp2r5ca*, *ppp2r5cb*, *slc8a1b*, *slc8a4a*, *slc9a1b*, *myh7*, *smyhc1*, *smyhc2*,	*slc8a4a*, *camk2a*, *cacng6b*	**―**
		**↓**	*ppp2cb*, *ppp2r2d*, *atp2b1a*, *atp2b3b*, *calm3a*, *prkcaa*, *creb1a*	*tpma*	**―**
**B.** Metabolism			***tirap*^-/-^ vs *tirap*^+/+^**	***myd88*^-/-^ vs *myd88*^+/+^**	***tlr2*^-/-^ vs *tlr2*^+/+^ 23**
Number of significantly altered metabolites			6	5	29
Amino acids and amines		**↑**	**―**	Serine, arginine	Glycine, 2-Aminobutyrate, kynurenine, 4-Aminobutyrate, leucine, asparagine, aspartate, threonine, valine, alanine, glutamine, tyrosine, ethanolamine
		**↓**	Proline, asparagine, serine, citrulline	Leucine, aspartate	Melatonin
Carbohydrates and organic acids		**↑**	**―**	2-Hydroxyglutarate	Succinate, 2-phosphoglycerate, glucose, indole-3-acetate, O-phosphoethanolamine, acetate, formate, galactose, glutamate, lactate, creatine, malate, taurine
		**↓**	2-Hydroxyglutarate, glucose	**―**	Glycerate
**C.** Leukocyte migration			***tirap*^-/-^ vs *tirap*^+/+^**	***myd88*^-/-^ vs *myd88*^+/+^**	***tlr2*^-/-^ vs *tlr2*^+/+^ 23**
Under unchallenged condition			No effect	No effect	No effect
Wounding response	Neutrophil	Recruitment	Increased	Reduced	Reduced
		Migration speed	Increased	No effect	No effect
		Directional persistence	No effect	Decreased meandering index	Decreased meandering index
	Macrophage	Recruitment	No effect	Reduced	Reduced
		Migration speed	No effect	Reduced	Reduced
		Directional persistence	No effect	Decreased meandering index	Decreased meandering index

The analysis of transcriptomic and metabolomic data for the *tirap* and *myd88* mutants is summarised from the current study, and that of *tlr2* mutant from previous study 22. Leukocyte migration studies for the *tirap* mutant were derived from the current study, and for *myd88* and *tlr2* from previous study 10. “↑” represents upward regulation or increased status compared with corresponding wild type control, “↓” represents downward regulation or reduced status compared with corresponding wild type control, “-” represents that no genes or metabolites were found. * represents that transcriptomic analysis of *tirap* and *myd88* mutants is based on padj value less than 0.05, whereas that of *tlr2* mutant is based on the s value less than 0.005

## References

[1] de Oliveira S, Rosowski EE, Huttenlocher A (2016). Neutrophil migration in infection and wound repair: going forward in reverse. Nat Rev Immunol.

[2] Hato T, Dagher PC (2015). How the Innate Immune System Senses Trouble and Causes Trouble. Clin J Am Soc Nephrol.

[3] Vijay K (2018). Toll-like receptors in immunity and inflammatory diseases: Past, present, and future. Int Immunopharmacol.

[4] Yamamoto M, Sato S, Hemmi H, Sanjo H, Uematsu S, Kaisho T (2002). Essential role for TIRAP in activation of the signalling cascade shared by TLR2 and TLR4. Nature.

[5] Lannoy V, Cote-Biron A, Asselin C, Rivard N (2023). TIRAP, TRAM, and Toll-Like Receptors: The Untold Story. Mediators Inflamm.

[6] Sampaio NG, Kocan M, Schofield L, Pfleger KDG, Eriksson EM (2018). Investigation of interactions between TLR2, MyD88 and TIRAP by bioluminescence resonance energy transfer is hampered by artefacts of protein overexpression. PLoS One.

[7] Bernard NJ, O'Neill LA (2013). Mal, more than a bridge to MyD88. IUBMB Life.

[8] Rajpoot S, Wary KK, Ibbott R, Liu D, Saqib U, Thurston TLM (2021). TIRAP in the Mechanism of Inflammation. Front Immunol.

[9] Filep JG (2021). Leukocytes in Inflammation, Resolution of Inflammation, Autoimmune Diseases and Cancer. Cells.

[10] Li L, Yan B, Shi YQ, Zhang WQ, Wen ZL (2012). Live imaging reveals differing roles of macrophages and neutrophils during zebrafish tail fin regeneration. J Biol Chem.

[11] Hu W, van Steijn L, Li C, Verbeek FJ, Cao L, Merks RMH (2021). A Novel Function of TLR2 and MyD88 in the Regulation of Leukocyte Cell Migration Behavior During Wounding in Zebrafish Larvae. Front Cell Dev Biol.

[12] Fitzgerald KA, Palsson-McDermott EM, Bowie AG, Jefferies C A, Mansell AS, Brady G, Brint E (2001). Mal (MyD88-adapter-like) is required for Toll-like receptor-4 signal transduction. Nature.

[13] Horng T, Barton GM, Flavell RA, Medzhitov R (2002). The adaptor molecule TIRAP provides signalling specificity for Toll-like receptors. Nature.

[14] Jeyaseelan S, Manzer R, Young SK, Yamamoto M, Akira S, Mason RJ (2005). Toll-IL-1 receptor domain-containing adaptor protein is critical for early lung immune responses against Escherichia coli lipopolysaccharide and viable Escherichia coli. J Immunol.

[15] Bernard NJ, Finlay CM, Tannahill GM, Cassidy JP, O'Neill LA, Mills KH (2015). A critical role for the TLR signaling adapter Mal in alveolar macrophage-mediated protection against Bordetella pertussis. Mucosal Immunol.

[16] Mansell A, Smith R, Doyle SL, Gray P, Fenner JE, Crack PJ (2006). Suppressor of cytokine signaling 1 negatively regulates Toll-like receptor signaling by mediating Mal degradation. Nat Immunol.

[17] Putranto EW, Murata H, Yamamoto K, Kataoka K, Yamada H, Futami J (2013). Inhibition of RAGE signaling through the intracellular delivery of inhibitor peptides by PEI cationization. Int J Mol Med.

[18] Sakaguchi M, Murata H, Yamamoto K, Ono T, Sakaguchi Y, Motoyama A (2011). TIRAP, an adaptor protein for TLR2/4, transduces a signal from RAGE phosphorylated upon ligand binding. PLoS One.

[19] Belhaouane I, Hoffmann E, Chamaillard M, Brodin P, Machelart A (2020). Paradoxical Roles of the MAL/Tirap Adaptor in Pathologies. Front Immunol.

[20] Meijer AH, Spaink HP (2011). Host-Pathogen Interactions Made Transparent with the Zebrafish Model. Curr Drug Targets.

[21] Renshaw SA, Loynes CA, Trushell DM, Elworthy S, Ingham PW, Whyte MK (2006). A transgenic zebrafish model of neutrophilic inflammation. Blood.

[22] Powell D, Tauzin S, Hind LE, Deng Q, Beebe DJ, Huttenlocher A (2017). Chemokine Signaling and the Regulation of Bidirectional Leukocyte Migration in Interstitial Tissues. Cell Rep.

[23] Hu W, Liu L, Forn-Cuni G, Ding Y, Alia A, Spaink HP (2023). Transcriptomic and Metabolomic Studies Reveal That Toll-like Receptor 2 Has a Role in Glucose-Related Metabolism in Unchallenged Zebrafish Larvae (Danio rerio). Biology (Basel).

[24] van der Vaart M, van Soest JJ, Spaink HP, Meijer AH (2013). Functional analysis of a zebrafish myd88 mutant identifies key transcriptional components of the innate immune system. Dis Model Mech.

[25] Livak KJ, Schmittgen TD (2001). Analysis of relative gene expression data using real-time quantitative PCR and the 2(-Delta Delta C(T)) Method. Methods.

[26] Hu W, Yang S, Shimada Y, Munch M, Marin-Juez R, Meijer AH (2019). Infection and RNA-seq analysis of a zebrafish tlr2 mutant shows a broad function of this toll-like receptor in transcriptional and metabolic control and defense to Mycobacterium marinum infection. BMC Genomics.

[27] Patro R, Duggal G, Love MI, Irizarry RA, Kingsford C (2017). Salmon provides fast and bias-aware quantification of transcript expression. Nat Methods.

[28] Love MI, Huber W, Anders S (2014). Moderated estimation of fold change and dispersion for RNA-seq data with DESeq2. Genome Biol.

[29] Zhu A, Ibrahim JG, Love MI (2019). Heavy-tailed prior distributions for sequence count data: removing the noise and preserving large differences. Bioinformatics.

[30] Ding Y, Haks MC, Forn-Cuni G, He J, Nowik N, Harms AC (2021). Metabolomic and transcriptomic profiling of adult mice and larval zebrafish leptin mutants reveal a common pattern of changes in metabolites and signaling pathways. Cell Biosci.

[31] Ding Y, Haks MC, van den Eeden SJF, Ottenhoff THM, Harms AC, Hankemeier T (2022). Leptin mutation and mycobacterial infection lead non-synergistically to a similar metabolic syndrome. Metabolomics.

[32] Benard EL, van der Sar AM, Ellett F, Lieschke GJ, Spaink HP (2012). Infection of Zebrafish Embryos with Intracellular Bacterial Pathogens. Journal of visualized experiments: JoVE.

[33] Stirling DR, Suleyman O, Gil E, Elks PM, Torraca V, Noursadeghi M (2020). Analysis tools to quantify dissemination of pathology in zebrafish larvae. Sci Rep.

[34] Santos-Sierra S, Deshmukh SD, Kalnitski J, Kuenzi P, Wymann MP, Golenbock DT (2009). Mal connects TLR2 to PI3Kinase activation and phagocyte polarization. EMBO J.

[35] Luan Y, Tang N, Yang J, Liu S, Cheng C, Wang Y (2022). Deficiency of ribosomal proteins reshapes the transcriptional and translational landscape in human cells. Nucleic Acids Res.

[36] Caterino M, Corbo C, Imperlini E, Armiraglio M, Pavesi E, Aspesi A (2013). Differential proteomic analysis in human cells subjected to ribosomal stress. Proteomics.

[37] Poddar D, Basu A, Baldwin WM 3rd, Kondratov RV, Barik S, Mazumder B (2013). An extraribosomal function of ribosomal protein L13a in macrophages resolves inflammation. J Immunol.

[38] Basu A, Poddar D, Robinet P, Smith JD, Febbraio M, Baldwin WM 3rd (2014). Ribosomal protein L13a deficiency in macrophages promotes atherosclerosis by limiting translation control-dependent retardation of inflammation. Arterioscler Thromb Vasc Biol.

[39] Slomiany BL, Slomiany A (2013). Involvement of p38 MAPK-dependent activator protein (AP-1) activation in modulation of gastric mucosal inflammatory responses to Helicobacter pylori by ghrelin. Inflammopharmacology.

[40] Guo RM, Xu WM, Lin JC, Mo LQ, Hua XX, Chen PX (2013). Activation of the p38 MAPK/NF-kappaB pathway contributes to doxorubicin-induced inflammation and cytotoxicity in H9c2 cardiac cells. Mol Med Rep.

[41] Baig MS, Liu D, Muthu K, Roy A, Saqib U, Naim A (2017). Heterotrimeric complex of p38 MAPK, PKCdelta, and TIRAP is required for AP1 mediated inflammatory response. Int Immunopharmacol.

[42] Sanin DE, Prendergast CT, Mountford AP (2015). IL-10 Production in Macrophages Is Regulated by a TLR-Driven CREB-Mediated Mechanism That Is Linked to Genes Involved in Cell Metabolism. J Immunol.

[43] Mellett M, Atzei P, Jackson R, O'Neill LA, Moynagh PN (2011). Mal mediates TLR-induced activation of CREB and expression of IL-10. J Immunol.

[44] Antony C, Mehto S, Tiwari BK, Singh Y, Natarajan K (2015). Regulation of L-type Voltage Gated Calcium Channel CACNA1S in Macrophages upon Mycobacterium tuberculosis Infection. PLoS One.

[45] Hosseini R, Lamers GEM, Bos E, Hogendoorn PCW, Koster AJ, Meijer AH (2021). The adapter protein Myd88 plays an important role in limiting mycobacterial growth in a zebrafish model for tuberculosis. Virchows Arch.

[46] Reiling N, Holscher C, Fehrenbach A, Kroger S, Kirschning CJ, Goyert S (2002). Cutting edge: Toll-like receptor (TLR)2- and TLR4-mediated pathogen recognition in resistance to airborne infection with Mycobacterium tuberculosis. J Immunol.

[47] Drennan MB, Nicolle D, Quesniaux VJ, Jacobs M, Allie N, Mpagi J (2004). Toll-like receptor 2-deficient mice succumb to Mycobacterium tuberculosis infection. Am J Pathol.

[48] Belhaouane I, Pochet A, Chatagnon J, Hoffmann E, Queval CJ, Deboosere N (2023). Tirap controls Mycobacterium tuberculosis phagosomal acidification. PLoS Pathog.

[49] Sobah ML, Liongue C, Ward AC (2021). SOCS Proteins in Immunity, Inflammatory Diseases, and Immune-Related Cancer. Front Med (Lausanne).

[50] Gleeson LE, Sheedy FJ, Palsson-McDermott EM, Triglia D, O'Leary SM, O'Sullivan MP (2016). Cutting Edge: Mycobacterium tuberculosis Induces Aerobic Glycolysis in Human Alveolar Macrophages That Is Required for Control of Intracellular Bacillary Replication. J Immunol.

[51] Lachmandas E, Beigier-Bompadre M, Cheng SC, Kumar V, van Laarhoven A, Wang X (2016). Rewiring cellular metabolism via the AKT/mTOR pathway contributes to host defence against Mycobacterium tuberculosis in human and murine cells. Eur J Immunol.

[52] Vicente-Manzanares M, Zareno J, Whitmore L, Choi CK, Horwitz AF (2007). Regulation of protrusion, adhesion dynamics, and polarity by myosins IIA and IIB in migrating cells. J Cell Biol.

[53] Doyle AD, Kutys ML, Conti MA, Matsumoto K, Adelstein RS, Yamada KM (2012). Micro-environmental control of cell migration-myosin IIA is required for efficient migration in fibrillar environments through control of cell adhesion dynamics. J Cell Sci.

[54] Bera K, Kiepas A, Godet I, Li Y, Mehta P, Ifemembi B (2022). Extracellular fluid viscosity enhances cell migration and cancer dissemination. Nature.

[55] Pillon M, Doublet P (2021). Myosins, an Underestimated Player in the Infectious Cycle of Pathogenic Bacteria. Int J Mol Sci.

[56] Mittal M, Siddiqui MR, Tran K, Reddy SP, Malik AB (2014). Reactive oxygen species in inflammation and tissue injury. Antioxid Redox Signal.

[57] Paredes-Zuniga S, Morales RA, Munoz-Sanchez S, Munoz-Montecinos C, Parada M, Tapia K (2017). CXCL12a/CXCR4b acts to retain neutrophils in caudal hematopoietic tissue and to antagonize recruitment to an injury site in the zebrafish larva. Immunogenetics.

[58] Robertson AL, Holmes GR, Bojarczuk AN, Burgon J, Loynes CA, Chimen M (2014). A zebrafish compound screen reveals modulation of neutrophil reverse migration as an anti-inflammatory mechanism. Sci Transl Med.

[59] Luo L, Lucas RM, Liu L, Stow JL (2019). Signalling, sorting and scaffolding adaptors for Toll-like receptors. J Cell Sci.

[60] Chen HG, Han WJ, Deng M, Qin J, Yuan D, Liu JP (2009). Transcriptional regulation of PP2A-A alpha is mediated by multiple factors including AP-2alpha, CREB, ETS-1, and SP-1. PLoS One.

