# Supplementary material for: Toll-like receptor adaptor protein TIRAP has specialized roles in signaling, metabolic control and leukocyte migration upon wounding in zebrafish larvae
Source: Int J Biol Sci. 2025 Jan 1;21(2):823–41. doi: 10.7150/ijbs.101055 (PMC11705633; doi:10.7150/ijbs.101055)
Supplement: Supplementary file 1 — Supplementary figures and tables. [file ijbsv21p0823s1.pdf]

Toll-like receptor adaptor protein TIRAP has specialized roles in signaling, metabolic control and leukocyte migration upon wounding in zebrafish larvae

Supplementary material

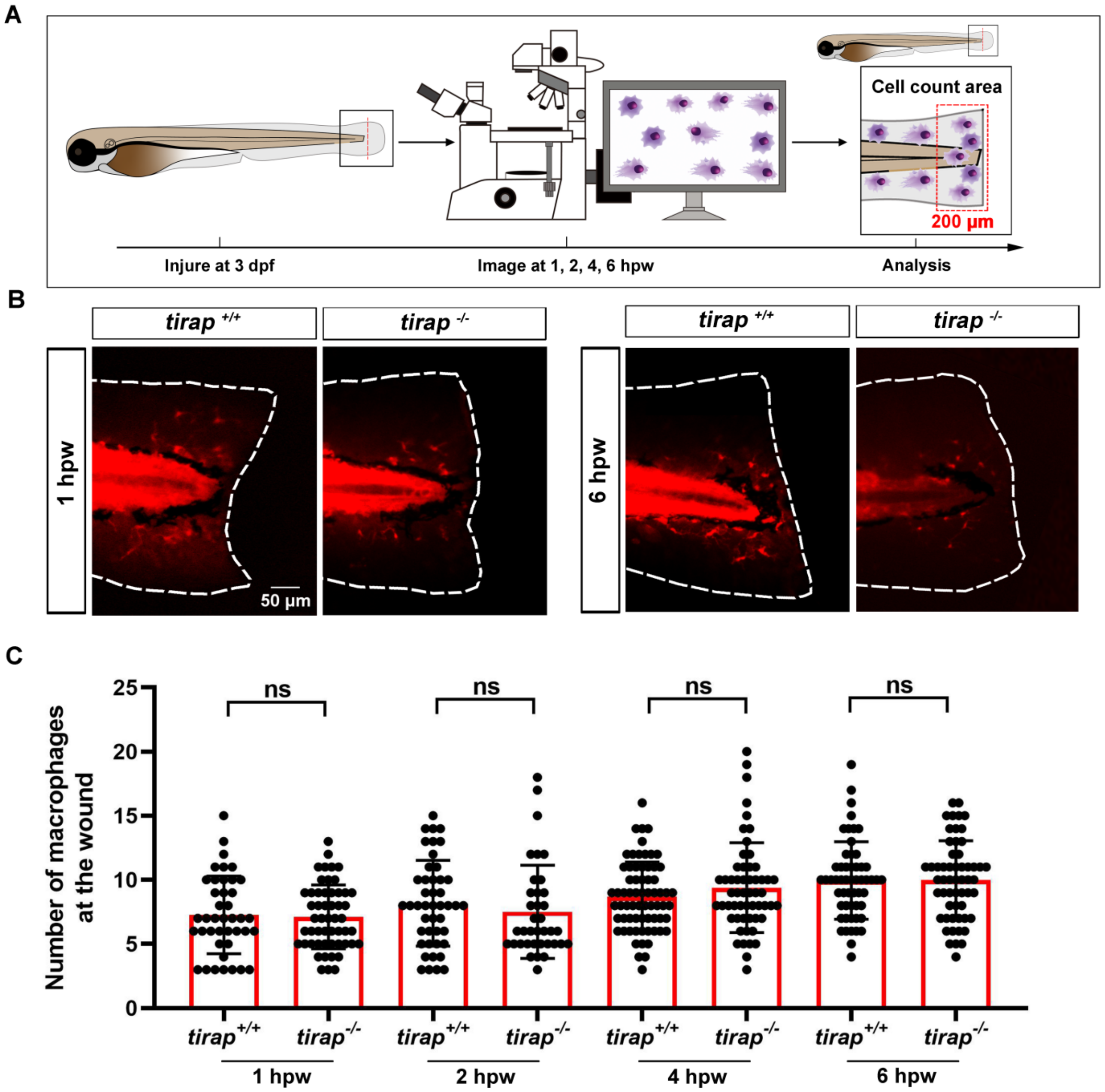

Figure S1. The number of recruited macrophages upon tail wounding in zebrafish larvae. (A) Schematic representation of experiment. The zebrafish larvae were imaged at designated times 1 hour post wounding (hpw), 2hpw, 4 hpw and 6 hpw. (B) Representative images of *tirap*$^{+/+}$ and *tirap*$^{-/-}$ at 1 hpw and 6 hpw. Scale bar: 50 µm. (C) Number of macrophages recruited to tail region upon tail wounding at 1, 2, 4, 6 hpw. The number of macrophages at 1 hpw from two independent experiments for *tirap*$^{+/+}$ (n=41) and *tirap*$^{-/-}$ (n=53). At 2 hpw for *tirap*$^{+/+}$ (n=45) and *tirap*$^{-/-}$ (n=37), at 4 hpw for *tirap*$^{+/+}$ (n=68) and *tirap*$^{-/-}$ (n=56) and at 6 hpw for *tirap*$^{+/+}$ (n=55) and *tirap*$^{-/-}$ (n=56) were based on three independent experiments. Statistical significant difference was determined by nonparametric tests: Mann-Whitney and Kolmogorov- Smirnov, ns, non-sginificant, *, $P<0.05$, **, $P<0.01$, ****, $P<0.0001$.

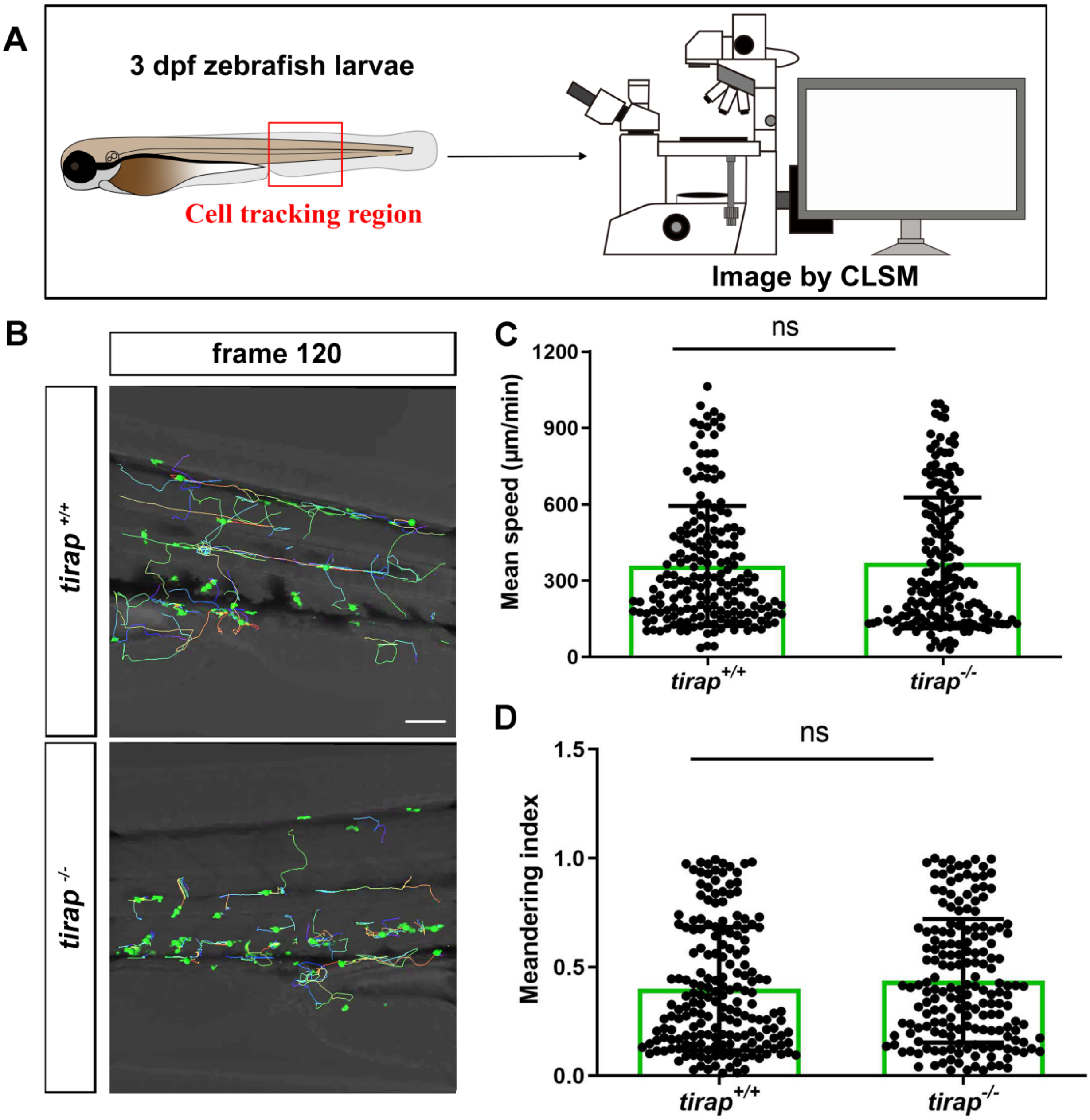

Figure S2. Quantification of neutrophil behaviour under unchallenged conditions. (A) Schematic representation of experiment. (B) Representative image of neutrophil behavior at frame 120. (C-D) Quantification of basal neutrophil migratory capability of 3 dpf *tirap* larvae at caudal hematopoietic tissue (CHT). Mean speed (C) and meandering index (D) were quantified using IMARIS Software (Imaris (RRID:SCR_007370)). The mean speed of neutrophils is measured as total displacement of neutrophils divided by the number of frames. Data is combined from 2 independent experiments for both *tirap*$^{+/+}$ (N=4) and *tirap*$^{-/-}$ (N=4). Statistical significant difference was determined by unpaired t test with Welch's correction, ns, non-significant.

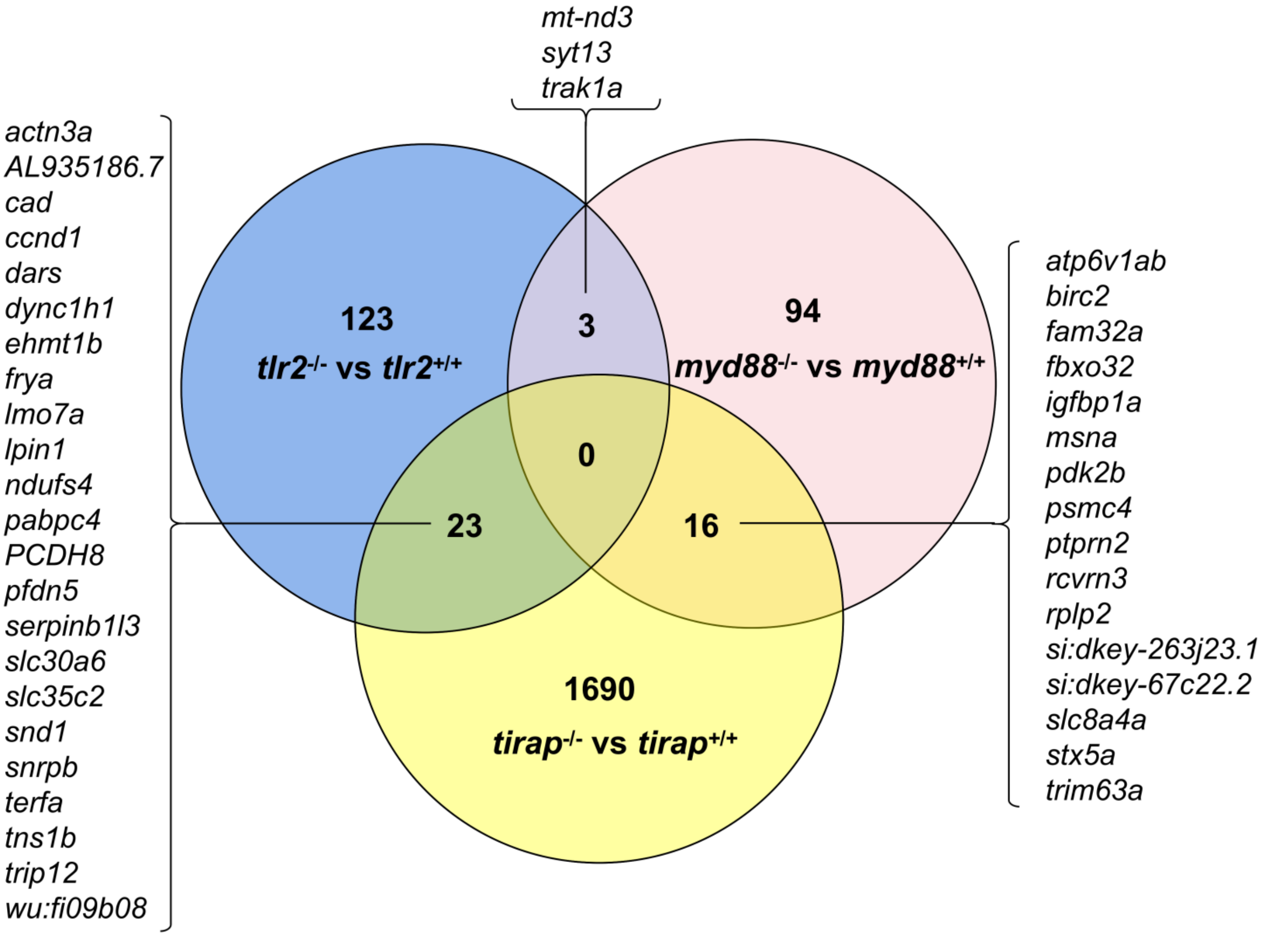

Figure S3. Venn diagram of DEGs from *tirap*$^{-/-}$ vs *tirap*$^{+/+}$ group, *tlr2*$^{-/-}$ vs *tlr2*$^{+/+}$ group and myd88$^{-/-}$ vs myd88$^{+/+}$ group. DEGs from *tirap*$^{-/-}$ vs *tirap*$^{+/+}$ group and myd88$^{-/-}$ vs myd88$^{+/+}$ group were assessed by padj value less than 0.05. DEGs from *tlr2*$^{-/-}$ vs *tlr2*$^{+/+}$ group were assessed by svalue less than 0.05 [23].

Table S1. The qRT-PCR primers used in this study

Primers	Sequences	Application
il1b-F	5'-GAACAGAATGAAGCACATCAAACC-3'	qRT-PCR
il1b-R	5'-ACGGCACTGAATCCACCAC-3'	qRT-PCR
il6-F	5′...TTCATGAGTCTCGCTGACCC...3′	qRT-PCR
il6-R	5′...GAGGAGTGCTGATCCTGACC...3′	qRT-PCR
il8-F	5′...TGTGTTATTGTTTTCCTGGCATTTC...3′	qRT-PCR
il8-R	5′...GCGACAGCGTGGATCTACAG...3′	qRT-PCR
il10-F	5′...GGAGACCATTCTGCCAACAGC...3′	qRT-PCR
il10-R	5′...TCTTGCATTTCACCATATCCCG...3′	qRT-PCR
tnfa-F	5′...AGACCTTAGACTGGAGAGATGAC...3′	qRT-PCR
tnfa-R	5′...CAAAGACACCTGGCTGTAGAC...3′	qRT-PCR
cebpb-F	5′...GCAGGCAACCTATCACCTACATAC...3′	qRT-PCR
cebpb-R	5′...CGCAAGTTTCACCGACTACAAGT...3′	qRT-PCR
fosl1a-F	5′...CTCAGCCCTCCCAATCACATCT...3′	qRT-PCR
fosl1a-R	5′...TACACTTCGCCGCAGCCATT...3′	qRT-PCR
ppial-F	5′...TGAGCCGCAACAGTAATC...3′	Reference
ppial-R	5′...AAGGGAAAAGGAAGTGAAAG...3′	Reference

Table S2. Differentially expressed genes (DEGs) in the representative pathway in *tirap*$^{-/-}$ versus *tirap*$^{+/+}$ zebrafish larvae groups.

Pathway	Ensembl ID	Gene name	log2 Fold Change	padj-Value	Description
TLR signaling	ENSDARG00000099184	*map2k6*	-1.510915968	0.000740717	mitogen-activated protein kinase kinase 6
	ENSDARG00000070573	*mapk3*	-1.668589839	4.00E-05	mitogen-activated protein kinase 3
	ENSDARG00000023110	*mapk7*	-1.376786107	0.015633315	mitogen-activated protein kinase 7
	ENSDARG00000042725	*cebpb*	-1.887025193	6.25E-05	CCAAT enhancer binding protein beta
	ENSDARG00000074378	*junba*	-1.628961987	0.003284556	JunB proto-oncogene, AP-1 transcription factor subunit a
	ENSDARG00000020133	*jdp2b*	-2.287062064	0.000234706	Jun dimerization protein 2b
	ENSDARG00000067850	*jund*	-1.188760964	0.012711251	JunD proto-oncogene, AP-1 transcription factor subunit
	ENSDARG00000037116	*cxcl12a*	-1.235618795	0.025460758	chemokine (C-X-C motif) ligand 12a (stromal cell-derived factor 1)
	ENSDARG00000004196	*tnfsf10l*	-1.24722621	0.02313739	TNF superfamily member 10, like
	ENSDARG00000098377	*tnfrsf11b*	7.489464879	0.023864106	tumor necrosis factor receptor superfamily, member 11b
	ENSDARG00000037553	*il1rapl2*	8.105000555	0.00416451	interleukin 1 receptor accessory protein-like 2
	ENSDARG00000074667	*akt1s1*	1.279698923	0.005125106	AKT1 substrate 1 (proline-rich)
	ENSDARG00000011219	*akt2*	1.2700915	0.00966533	v-akt murine thymoma viral oncogene homolog 2
	ENSDARG00000087205	*akt3b*	8.297200628	1.50E-05	v-akt murine thymoma viral oncogene homolog 3b
Glycolysis and TCA cycle	ENSDARG00000060504	*pfkla*	6.037617359	0.02513267	phosphofructokinase, liver a
	ENSDARG00000054191	*pgk1*	1.065360736	0.023951103	phosphoglycerate kinase 1
	ENSDARG00000039093	*got1*	1.463607063	0.02926267	glutamic-oxaloacetic transaminase 1, soluble
	ENSDARG00000042010	*pklr*	-1.376344872	0.00580824	pyruvate kinase L/R
	ENSDARG00000059054	*pdk2b*	1.197079899	0.046865975	pyruvate dehydrogenase kinase, isozyme 2b
	ENSDARG00000076308	*pdp1*	7.881587716	0.014198245	pyruvate dehyrogenase phosphatase catalytic subunit 1
	ENSDARG00000043371	*mdh2*	0.960102902	0.036123462	malate dehydrogenase 2, NAD (mitochondrial)
	ENSDARG00000005359	*sucla2*	1.140335947	0.048819898	succinate-CoA ligase, ADP-forming, beta subunit
	ENSDARG00000103364	*cs*	1.082934649	0.004366919	citrate synthase
	ENSDARG00000055652	*aclyb*	-2.434685739	0.025909141	ATP citrate lyase b
Autophagy	ENSDARG00000040657	*wipi1*	-1.401387052	0.033030684	WD repeat domain, phosphoinositide interacting 1
	ENSDARG00000075139	*hdac5*	-2.190324537	0.031243992	histone deacetylase 5
	ENSDARG00000086458	*hdac10*	-1.105818447	0.035662922	histone deacetylase 10

	ENSDARG00000062518	*ulk1a*	9.354760047	5.73E-07	unc-51 like autophagy activating kinase 1a
	ENSDARG00000040277	*fbxo32*	1.768701643	0.003885178	F-box protein 32
	ENSDARG00000055292	*atg9b*	1.368894618	0.007208796	autophagy related 9B
	ENSDARG00000052818	*smcr8a*	7.776146188	0.038195224	Smith-Magenis syndrome chromosome region, candidate 8a
	ENSDARG00000097245	*soga3a*	9.984814829	2.00E-08	SOGA family member 3a
	ENSDARG00000037871	*wipi2*	2.352236748	1.07E-05	WD repeat domain, phosphoinositide interacting 2
	ENSDARG00000037560	*mtm1*	6.044536193	0.001407213	myotubularin 1
	ENSDARG00000056842	*usp30*	7.613615523	0.016816102	ubiquitin specific peptidase 30
	ENSDARG00000036338	*vps11*	1.562483319	0.013571365	vacuolar protein sorting 11
Ribosome	ENSDARG00000053457	*rpl23*	-1.31832882	0.011619987	ribosomal protein L23
	ENSDARG00000099104	*rpl24*	-1.221610002	0.031143469	ribosomal protein L24
	ENSDARG00000005791	*rpl28*	-1.221797114	0.027097835	ribosomal protein L28
	ENSDARG00000077717	*rpl29*	-1.367320533	0.002203954	ribosomal protein L29
	ENSDARG00000035871	*rpl30*	-1.211077303	0.023579704	ribosomal protein L30
	ENSDARG00000036316	*rpl39*	-1.09012718	0.048576788	ribosomal protein L39
	ENSDARG00000011201	*rplp2l*	-1.218283147	0.023013393	ribosomal protein, large P2, like
	ENSDARG00000036298	*rps13*	-1.340170625	0.014103277	ribosomal protein S13
	ENSDARG00000030602	*rps19*	-1.174866323	0.041930897	ribosomal protein S19
	ENSDARG00000025850	*rps21*	-1.109696313	0.021019913	ribosomal protein S21
	ENSDARG00000041811	*rps25*	-1.219167475	0.027363843	ribosomal protein S25
	ENSDARG00000037071	*rps26*	-1.167312016	0.026665233	ribosomal protein S26
	ENSDARG00000041232	*rps29*	-1.222203934	0.014053931	ribosomal protein S29
Oxidative phosphorylation	ENSDARG00000052840	*ndufs4*	-1.151745645	0.038414796	NADH:ubiquinone oxidoreductase subunit S4
	ENSDARG00000042469	*ndufa12*	-1.559292267	0.010102699	NADH:ubiquinone oxidoreductase subunit A12
	ENSDARG00000045230	*cox6b1*	-1.088023429	0.014277173	cytochrome c oxidase subunit 6B1
	ENSDARG00000027355	*slc25a4*	1.220084402	0.048494089	solute carrier family 25 (mitochondrial carrier; adenine nucleotide translocator), member 4
Calcium regulation	ENSDARG00000042552	*cacna1sb*	1.391654176	0.02922106	calcium channel, voltage-dependent, L type, alpha 1S subunit, b
	ENSDARG00000078169	*cacna2d2b*	8.211738729	0.00663626	calcium channel, voltage-dependent, alpha 2/delta subunit 2b
	ENSDARG00000063006	*cacng7a*	7.349292843	0.034747364	calcium channel, voltage-dependent, gamma subunit 7a
	ENSDARG00000099241	*ppp2cb*	-1.464703532	0.004401995	protein phosphatase 2, catalytic subunit, beta isozyme

ENSDARG00000102009	*ppp2r2d*	-2.224775103	9.14E-07	protein phosphatase 2, regulatory subunit B, delta
ENSDARG00000059083	*ppp2r5ca*	2.239352665	0.006191032	protein phosphatase 2, regulatory subunit B', gamma a
ENSDARG00000031200	*ppp2r5cb*	1.32778729	0.01328537	protein phosphatase 2, regulatory subunit B', gamma b
ENSDARG00000043406	*slc8a1b*	9.089887066	1.58E-05	solute carrier family 8 (sodium/calcium exchanger), member 1b
ENSDARG00000055154	*slc8a4a*	7.727366289	0.015953285	solute carrier family 8 (sodium/calcium exchanger), member 4a
ENSDARG00000097339	*slc9a1b*	7.833197174	0.008092416	solute carrier family 9 member A1b
ENSDARG00000012684	*atp2b1a*	-1.571028762	0.00190273	ATPase plasma membrane Ca2+ transporting 1a
ENSDARG00000023445	*atp2b3b*	-2.237791493	5.07E-08	ATPase plasma membrane Ca2+ transporting 3b
ENSDARG00000079564	*myh7*	1.452430669	0.029242822	myosin heavy chain 7
ENSDARG00000099959	*smyhc1*	0.974799495	0.041781027	slow myosin heavy chain 1
ENSDARG00000103969	*smyhc2*	2.241942444	4.94E-07	slow myosin heavy chain 2
ENSDARG00000100825	*calm3a*	-1.809712548	2.21E-06	calmodulin 3a (phosphorylase kinase, delta)
ENSDARG00000115657	*prkcaa*	-1.077143247	0.010520081	protein kinase C, alpha, a
ENSDARG00000033184	*prkar2aa*	-1.041567526	0.034767684	protein kinase, cAMP-dependent, regulatory, type II, alpha A
ENSDARG00000053586	*creb1a*	-1.357761461	0.021504733	cAMP responsive element binding protein 1a

Table S3. Differentially expressed genes (DEGs) in the representative pathway in *myd88*$^{-/-}$ versus *myd88*$^{+/+}$ zebrafish larvae groups.

Pathway	Ensembl ID	Gene name	log2 Fold Change	padj-Value	Description
TLR signaling	ENSDARG00000006409	*mapk12b*	4.820780436	0.022554316	mitogen-activated protein kinase 12b
Glycolysis and TCA cycle	ENSDARG00000020364	*fbp1b*	1.753309151	0.036384064	fructose-1,6-bisphosphatase 1b
	ENSDARG00000059054	*pdk2b*	1.938947554	5.01E-06	pyruvate dehydrogenase kinase, isozyme 2b
Ribosome	ENSDARG00000112656	*rpl36a*	-1.469863763	0.013985859	ribosomal protein L36A
	ENSDARG00000006413	*rpl38*	-1.472779904	0.019617391	ribosomal protein L38
	ENSDARG00000101406	*rplp2*	-1.394220944	0.040024964	ribosomal protein, large P2
Oxidative phosphorylation	ENSDARG00000076318	*atp6v1ab*	1.732618721	0.000799351	ATPase H+ transporting V1 subunit Ab
	ENSDARG00000063914	*mt-nd3*	-1.350342543	0.025536145	NADH dehydrogenase 3, mitochondrial
	ENSDARG00000110786	*ndufb4*	-9.567162039	5.37E-08	NADH:ubiquinone oxidoreductase subunit B4
	ENSDARG00000103101	*ndufc1*	-1.588330341	0.020847424	si:ch211-235e9.6
	ENSDARG00000069464	*cox7a1*	-2.271411629	0.002852318	cytochrome c oxidase subunit 7A1
Calcium regulation	ENSDARG00000055154	*slc8a4a*	6.94081136	0.010673858	solute carrier family 8 (sodium/calcium exchanger), member 4a
	ENSDARG00000053617	*camk2a*	4.238435243	0.044672365	calcium/calmodulin-dependent protein kinase II alpha
	ENSDARG00000046079	*cacng6b*	1.865932062	0.017048177	calcium channel, voltage-dependent, gamma subunit 6b
	ENSDARG00000033683	*tpma*	-1.398430746	0.012743848	alpha-tropomyosin

Table S4. 6 significantly altered metabolites in *tirap*$^{-/-}$ versus *tirap*$^{+/+}$ zebrafish larvae groups.

HMDB ID	Metabolite name	log2 Fold Change	*P* value
HMDB0000162	Proline	-0.972289172	0.0017707
HMDB0059655	2-Hydroxyglutarate	-0.933348095	0.00534
HMDB0000191	Asparagine	-3.248860793	0.0056096
HMDB0000122	Glucose	-0.998747512	0.0071217
HMDB0000187	Serine	-4.390942773	0.016276
HMDB0000904	Citrulline	-2.326894348	0.022583

Table S5. 5 significantly altered metabolites in *myd88*$^{-/-}$ versus *myd88*$^{+/+}$ zebrafish larvae groups.

HMDB ID	Metabolite name	log2 Fold Change	*P* value
HMDB0000187	Serine	1.374635145	0.039828
HMDB0000517	Arginine	0.61653258	0.014019
HMDB0059655	2-Hydroxyglutarate	0.552629638	0.0090725
HMDB0000191	Aspartate	-0.562953575	0.023516
HMDB0000687	Leucine	-2.106980756	0.03105
